# *Persea americana* Peel: A Promising Source of Nutraceutical for the Mitigation of Cardiovascular Risk in Arthritic Rats Through the Gut–Joint Axis

**DOI:** 10.3390/biom15040590

**Published:** 2025-04-16

**Authors:** Doha A. Mohamed, Asmaa A. Ramadan, Hoda B. Mabrok, Gamil E. Ibrahim, Shaimaa E. Mohammed

**Affiliations:** 1 Nutrition and Food Science Department, Food Industries and Nutrition Institute, National Research Centre, Cairo 12622, Egypt; ae.ramadan@nrc.sci.eg (A.A.R.); hoda.mabrok@gmail.com (H.B.M.); shaimaa.elsayed1876@gmail.com (S.E.M.); 2 Chemistry of Aroma and Flavor Department, Food Industries and Nutrition Institute, National Research Centre, Cairo 12622, Egypt; gamilemad2000@gmail.com

**Keywords:** avocado peel, nutraceutical, gut–joint axis, rheumatoid arthritis, cardiovascular diseases, gene expression

## Abstract

Rheumatoid arthritis (RA) is a chronic autoimmune inflammatory disease characterized by the inflammation of synovial fluid. The incidence of cardiovascular diseases (CVDs) is increasing in RA patients. This research is the first report to investigate the anti-arthritic effect of avocado peel nutraceutical (APN) and its potential in mitigating the cardiovascular risk associated with RA. The antioxidant activity and phytochemical composition of APN were assessed. The potential interaction of APN’s active compounds with protein tyrosine phosphatase non-receptor type 22 (PTPN22) was studied using molecular docking. The impact of APN on the plasma lipid profile, oxidative and inflammatory markers, and the indices of coronary risk and atherogenicity as CVD markers were evaluated. The gene expression of COX-2, IL-6, IL-1β, IL-10, and TNF-α in liver and spleen tissues were measured. The rat gut microbiota profile was investigated using 16S rRNA amplicon sequencing. APN exhibited high antioxidant activity, low atherogenicity and thrombogenicity indices, and a high ratio of hypocholesterolemic to hypercholesterolemic fatty acids indicating its cardioprotective potential. The administration of APN led to a reduction in oxidative stress markers, inflammatory markers, dyslipidemia, and CVD markers. APN administration downregulated the expression of COX-2, IL-6, IL-1β, and TNF-α genes, while the IL-10 gene was significantly upregulated in the liver and spleen. Treatment with APN was favorable in restoring eubiosis in the gut by modulating RA-associated bacterial taxa linked to impaired immune function and cardiometabolic diseases. In molecular docking, β-amyrin and ellagic acid showed the highest binding affinity for PTPN22. APN may represent a promising approach to ameliorating the cardiovascular risk of RA. The present results will be offering a foundation for future in-depth research in nutraceuticals from agriculture by-products. Additionally, they will be supporting the public health policies aimed at preventing and controlling rheumatoid arthritis.

## 1. Introduction

Immune-mediated inflammatory diseases (IMIDs) encompass a variety of chronic disorders characterized by unusual immune responses and tissue inflammation [1]. Rheumatoid arthritis (RA) is one such IMID, distinguished by symmetric pain in small joints, stiffness, tenderness, and swelling [2]. Early symptoms commonly affect the hands and feet, particularly across the metacarpophalangeal and metatarsophalangeal joints. RA affects approximately 1% of adults aged 20–40 years worldwide and is more prevalent in individuals over 75 years old [3,4]. Inflammation plays a critical role in RA leading to joint damage, deformities, and disability. It also contributes to the elevation of inflammatory cytokines such as IL-1, IL-6, and TNF-α, which induce endothelial dysfunction [5]. RA is associated with numerous complications including cardiovascular diseases (CVDs), osteoporosis, and hypertension. The incidence of CVDs in RA patients has been reported to be 1.48-fold higher than in healthy individuals. CVDs are among the most common complications of RA patients and a leading cause of mortality. Inflammation and oxidative stress are key mechanisms underlying the increased incidence of CVDs in RA patients, driven by alterations in lipid profiles, lipoprotein profiles (LDL & HDL), and the oxidation of LDL [6,7].

Gut microbiota plays a crucial role in maintaining immune homeostasis. Disruptions in gut microbial composition and metabolite production can alter immune cell responses, contributing to the development of IMIDs such as inflammatory bowel diseases and rheumatoid arthritis [8,9]. Gut dysbiosis has been observed in individuals at high risk for RA [10], and the severity of RA has been correlated with dysbiosis [2,11]. Certain gut bacterial taxa have been linked to the induction of autoimmune responses in arthritis-susceptible mouse models. Dysbiosis may activate the innate immune system, exacerbating RA through the gut–joint axis. The proposed mechanism involves alterations in the metabolism of specific gut bacteria, which compromise the host immune profile through bacterial metabolites that regulate immune function [11,12,13,14,15,16].

Avocado (*Persea americana* L., Family Lauraceae) is a tropical fruit [17] primarily consumed fresh and used in oil production [18]. The processing of avocado generates significant quantities of by-products, such as seeds and peels [19]. Avocado peels account for approximately 16% of the fruit’s dry weight [20]. Utilizing these waste materials by extracting their phytochemical content could lead to the development of novel beneficial products and add value to the avocado industry [21]. Avocado by-products are rich sources of nutrients including proteins, healthy fats, and phytochemical compounds such as volatile compounds, phenolic compounds, condensed tannins, triterpenoids, phytosterols, and polyphenolics [22,23]. They are also abundant in lipophilic antioxidants (e.g., phytosterols, acetogenins, and monounsaturated fatty acids), which can penetrate cell membranes and exhibit superior bioavailability compared to hydrophilic antioxidants [24]. The bioactive compounds in avocado by-products have demonstrated anti-inflammatory, antioxidant, hypoglycemic, and cardiovascular protective activities [25]. These compounds enhance cardiovascular health by reducing blood pressure and improving endothelial function [26]. Therefore, the utilization of avocado waste as a source of active compounds against chronic diseases aligns with sustainable development and environmental responsibility.

Despite these findings, to the best of our knowledge, no previous research has evaluated the effect of avocado peel on rheumatoid arthritis. Furthermore, no nutraceutical derived from avocado peel has been developed for the treatment of RA. Therefore, the objective of this study was to prepare an avocado peel nutraceutical (APN) as a rich source of phytochemicals and evaluate its anti-arthritic effect and potential to mitigate cardiovascular diseases in arthritic rats. The impact of APN on the expression of cyclooxygenase-2 (COX-2), interleukin-6 (IL-6), interleukin-1β (IL-1β), interleukin-10 (IL-10), and tumor necrosis factor-α (TNF-α) genes was assessed in liver and spleen tissues. Additionally, oxidative and inflammatory markers in rat plasma were evaluated. The indices of coronary risk and atherogenicity were used as CVD markers. The gut microbiota profile of arthritic rats was investigated using 16S rRNA amplicon sequencing to assess the involvement of the gut–joint-axis. Finally, the potential interaction between APN’s active compounds and protein tyrosine phosphatase non-receptor type 22 (PTPN22) was examined through molecular docking studies.

## 2. Materials and Methods

### 2.1. Plant Material

Avocado fruits (*Persea americana* Mill., Lauraceae) were obtained from the Egyptian local market (PICO Modern Agriculture Company, Zamalek, Egypt). The fruits of avocado were rinsed by tap water, peeled, and then the peel was thinly sliced and freeze-dried.

### 2.2. Animals

Male Wister rats of 105.83 ± 4.3 g as mean ± SD were brought from the veterinary animal house of the National Research Centre, Cairo, Egypt. The rats were individually housed in stainless steel cages under standard lab conditions (23–25 °C, 12 h light/dark cycle) with full access to food and water. This study was conducted as part of project No. 13050203 funded by the NRC. The project procedures were approved by the Medical Research Ethics Committee, NRC (approval number 13050203) following the recommendations of the National Institutes of Health Guide for Care and Use of Laboratory Animals (Publication No. 85-23, revised 1985).

### 2.3. Extraction and Preparation of Avocado Peel Nutraceutical

Freeze-dried avocado peel powder was subjected to oil extraction using petroleum ether at 40–60 °C. For the extraction of polar bioactive phytochemicals, such as phenolic compounds, ethanol—a GRAS (Generally Recognized As Safe) solvent—was employed using ultrasound-assisted extraction. Both solvents were completely evaporated under vacuum. The ethanol extract and petroleum ether extract (oil) from avocado peel were then combined at a 1:1 ratio to prepare the APN.

### 2.4. Screening of the Active Compounds and Antioxidant Activity of Avocado Peel Nutraceutical

APN was analyzed to determine the content of various bioactive compounds, including total phenolic compounds using the Folin–Ciocalteu method [27] and total flavonoids [28]. The results for total phenolic and total flavonoids were expressed as mg gallic acid equivalent per gram of dried sample (mg GAE/g) and mg catechin equivalent per gram of dried sample (mg CE/g), respectively. The phenolic compound profile of APN was determined using HPLC (Agilent 1260 series) and under the same condition reported by Mohamed et al. [29]. The concentration of each phenolic compound was identified by comparison with a standard mixture containing gallic acid, methyl gallate, chlorogenic acid, catechin, pyrocatechol, rosmarinic acid, syringic acid, rutin, coumaric acid, coffeic acid, naringenin, ferulic acid, vanillin, cinnamic acid, daidzein, querectin, hesperetin, and kaempferol. For the determination of volatile compounds, APN was reconstituted in 1 mL of methanol/water (50:50, *v*/*v*). The extracts were then filtered through regenerated cellulose filters 0.45 μm (Millipore, Bedford, MA, USA) and stored at −18 °C until analysis. Volatile compounds were extracted and identified using solid-phase microextraction (SPME) [30] and the methods described by Adams [31]. Fatty acid methyl esters, triterpenoids, and phytosterols in APN were prepared according to AOAC standard procedures [32] and analyzed using GLC. Assessment of the methyl ester was carried out by injecting 2 µL into a Hewlett Packard HP-system 6890 gas chromatograph equipped with FID. HP-5 capillary column (30 m × 0.32 mm i.d.; 0.25 µm film thickness) was used to separate the different methyl esters. The chromatographic analysis conditions were an initial temperature of 70 °C with a hold for 1 min, then raised to 120 °C at a rate of 40 °C/min with 2 min hold; then, the temperature was finally raised to 220 °C at a rate of 4 °C/min with another 20 min hold. The injector and detector temperatures were 250 °C and 280 °C, respectively. Identification of the fatty acid methyl esters was carried out by a direct comparison of retention times of each of the separated compounds with standards of the fatty acid methyl esters analyzed under the same conditions. Quantization was based on peak area integration. The assessment and identification of triterpenoids and phytosterols were performed under the same conditions described by Mohamed et al. [33]. The nutritional quality indices and oxidizability (COX) value of APN were calculated using Formula (1) from Fatemi and Hammond [34]. Nutritional quality indices, including the thrombogenicity index (TI), atherogenicity index (AI), and the ratio of hypocholesterolemic/hypercholesterolemic (H/H) fatty acids, were computed according to Formulas (2)–(4), respectively, as reported by Ulbricht and Southgate [35]. The antioxidant activity of APN was evaluated using the DPPH and ABTS methods as previously described by Brand-Williams et al. [36] and Re et al. [37], respectively. The results of the antioxidant activity were calculated as Trolox equivalents (TE).

COX = C18:1 + 10.3 × C18:2 + 21.6 × C18:3 100(1)

AI = (C12:0 + 4 × C14:0 + C16:0)/(∑MUFA + ∑(ω − 3)+ ∑(ω − 6))(2)

TI = (C14:0 + C16:0 + C18:0)/(0.5 × MUFA + 0.5 × ∑(ω − 6) + 3 × ∑(ω − 3) + (ω − 3/ω − 6))(3)

H/H = (C18:1 + C18:2 + C18:3 + C18:4 + C20:4)/(C14:0 + C16:0)(4)

### 2.5. Evaluation of the Anti-Arthritic Effect of APN and Its Potential for the Mitigation of Cardiovascular Risk

**Figure 1:**
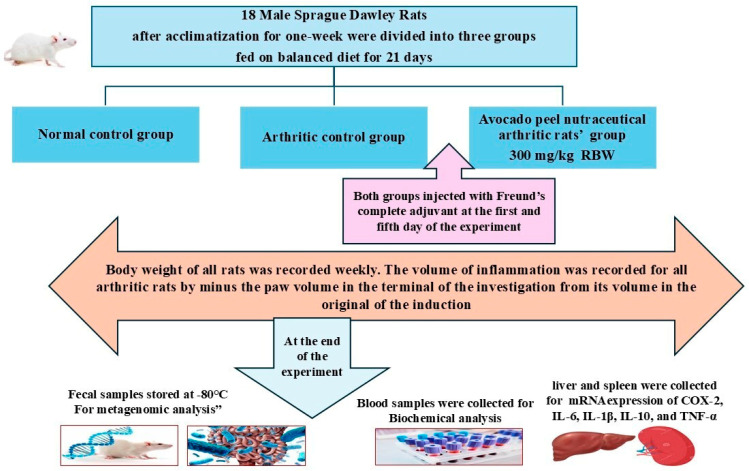
Flow chart of the animal experiment.

The anti-arthritic potential of APN was evaluated in rats following the procedure described by Hassan et al. [38], using Freund’s complete adjuvant (FCA) (Sigma-Aldrich, St. Louis, USA) as the arthritis inducer. Eighteen rats were divided into three groups (*n* = 6 per group). Group I served as the normal control, while group II served as the arthritic control. APN was prepared in the form of water suspension using Tween 80 as a suspending agent to be orally administered to rats. Group III was administered APN as a daily oral dose of 300 mg/kg rat body weight for three weeks. All groups were fed a balanced diet throughout the study period. On the second day of the study, all groups except the normal control were injected with FCA into the subplantar region of the right hind paw [38]. To increase the severity of arthritis, 0.1 mL of FCA was injected into the paw five days after the first injection in all rats except the normal control. The inflamed paws of all arthritic rats were measured before the induction of arthritis and at the end of the study using vernier calipers. The inflammation volume (mm) was calculated for all arthritic rats by subtracting the initial paw volume from the volume at the end of the experiment. Figure 1 illustrates the flow chart of the animal experimental design. Fecal samples were collected from all animals and immediately stored at −80 °C until metagenomic analysis. At the end of the study, blood samples were collected from all groups after an overnight fast for biochemical analysis. Following anesthesia and euthanasia, the liver and spleen were dissected from all rats to study the mRNA expression of COX-2, IL-6, IL-1β, IL-10, and TNF-α.

### 2.6. Biochemical Analysis of Blood Samples of Different Experimental Groups

Blood samples were collected from fasting rats at the end of the experiment for the determination of plasma tumor necrosis factor-α (TNF-α) (ELISA kit, Catalogue # SL0722Ra, Sunlong^®^, AChE) in brain tissue), Interleukin 1-β (IL-1-β) (ELISA kit, Catalogue # SL0042Ra, Sunlong^®^, AChE) in brain tissue), and Interleukin-6 (IL-6) (ELISA kit, Catalogue # SL0411Ra, Sunlong^®^, AChE) in brain tissue) as inflammatory markers. Malondialdehyde (MDA) [39] and catalase activity [40] were evaluated as indicators of oxidative stress. Plasma lipid profile parameters (total cholesterol (T-Ch), triglycerides (TG), and high-density lipoprotein cholesterol (HDL-Ch)) were determined using colorimetric kits, while oxidized-LDL (oxi-LDL) was determined using ELISA kit (Catalogue # SL0554Ra, Sunlong^®^, AChE) in brain tissue). Low-density lipoprotein cholesterol (LDL-Ch) was calculated according to the formula (LDL-Ch = T-Ch-HDL-Ch (TG/5)). The ratios of T-Ch/HDL-Ch and LDL-Ch/HDL-Ch were calculated as coronary risk index and atherogenic index, respectively. Plasma levels of urea and creatinine were determined as indicators of kidney function, while aspartate transaminase (AST) and alanine transaminase (ALT) were evaluated as markers of liver function using colorimetric kits.

### 2.7. mRNA Expression of IL-6, IL-1β, IL-10, TNF-α, and COX-2

Total RNA was extracted from the liver and spleen tissue by PureLink^®^RNA Mini-Kit (ambion^®^Lifetechnologies^TM^, California, USA) according to the supplied protocol. The purity and concentration of RNA were determined with a Nano-Drop spectrophotometer. According to the cDNA synthesis procedure, a TOPscript™RT dry mix Kit (enzynomics™, Daejeon, Republic of Korea) was used for the reverse transcription of total RNA according to the given protocol. Primer’s sequence used for Cyclooxygenase-2 (COX-2), Interleukin-6 (IL-6), Interleukin-1β (IL-1β), Interleukin-10 (IL-10), and TNF-α gene expression analysis are provided in Table 1. Real-Time PCR was performed in a Rotor-Gene^®^MDx instrument (Qiagen, Düsseldorf, Germany). cDNA as template was amplified by EVA-Green Master Mix (HOT FIREPolEvaGreen qPCR Mix Plus, Solis BioDyne^TM^, Tartu, Estonia) in 20 µL reaction mixture. The reaction mixture was heated for 2 min at 50 °C, then at 95 °C for 12 min; then, it was followed by 45 cycles including 95 °C for 20 s, 60 °C for 30 s, and 72 °C for 20 s. The final step was plotting the melting curve program (from 60 to 95 °C). The relative expression of target genes was computed using the 2^−∆∆CT^ method [41]. Gene expression levels of COX-2, IL6, IL1β, IL10, and TNFα were normalized to the house-keeping gene GAPDH (Table 1).

### 2.8. 16S rRNA Amplicon Sequencing Analysis of Colon Content

Genomic DNA was extracted from colon content samples using (EasyPure^®^ stool genomic DNA kit, Transgen Biotech, Beijing, China) according to the maufacturer’s protocol. Next generation sequencing of 16S rRNA took place at BMKGENE (Beijing, China). Illumina Novaseq 6000 workstation (Illumina, San Diego, CA, USA) was used to sequence the V3 and V4 regions of the bacterial 16S rRNA gene.

The raw reads were filtered and trimmed using Trimmomatic v 0.33. Primer sequence identification and removal was processed by Cutadapt (version 1.9.1). Paired-end reads were assembled by USEARCH (version 10), and chimeric structures were removed using UCHIME (version 8.1). Finally, the resulting sequences with similarity over 97% were clustered into operational taxonomic units (OTUs) according to Edgar [44], with a conservative threshold of 0.005% for OTU filtration according to Bokulich et al. [45]. Taxonomic annotation of feature sequences was performed by Bayesian classifier using SILVA reference database, Release138.

Abundances of different taxa, alpha diversity indices including ACE, Simpson, and Shannon, as well as PCoA analysis (Beta diversity) were calculated. The community composition of each group at multiple levels of classification including phylum, family, and genus was determined. Significant differences in abundance between groups were analyzed using ANOVA and *p*-values were corrected for multiple comparisons using the Benjamini and Hochberg-FDR methods. Differential analysis, correlation analysis, and functional prediction were performed. Bioinformatics analysis was performed on the BMK-Cloud platform.

### 2.9. Molecular Docking Studies of Avocado Peel Nutraceutical

The protein tyrosine phosphatase non-receptor type 22 (PTPN22) structure was retrieved from the UniProt database. The protein structures were prepared using AutoDock Tools 1.5.7, including addition of hydrogen atoms, removal of H_2_O molecules, and assignment of partial charges. The structures of major phytochemicals present in avocado peel were obtained from the PubChem database. The retrieved compounds were energy minimized using Avogadro molecular modeling software (version 1.2.0) and the MMFF94 force field. Molecular docking was performed using AutoDock Vina. The prepared protein and ligands were used as inputs for the docking process. The docking results were visualized using the BIOVIA software, version 2020 [29].

### 2.10. Statistical Analysis

The SPSS version 26 statistical program was used to analyze the data using one-way ANOVA followed by Tukey’s multiple comparison test. Statistical significance was determined at a significance level of *p* ≤ 0.05.

## 3. Results

### 3.1. Screening of the Active Compounds of Avocado Peel Nutraceutical and Its Antioxidant Activity

**Figure 2:**
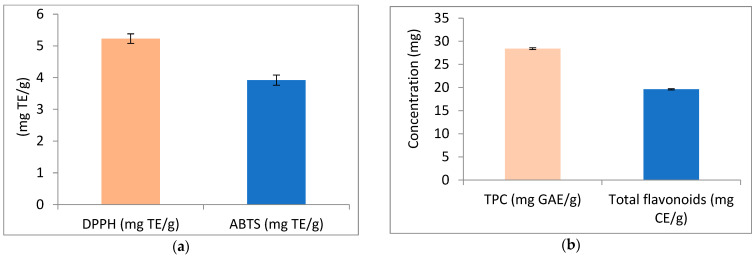
Antioxidant activity, phenolic compounds (TPC), and flavonoids of avocado peel nutraceutical. (**a**) DPPH and ABTS activity of APN as mg/Trolox Equivalent (TE); (**b**) phenolic compounds and flavonoids content in APN.

The results of antioxidant activity, total phenolics, and total flavonoids of APN are illustrated in Figure 2. Figure 2a represents the antioxidant activity of APN using the DPPH and ABTS methods. The results revealed antioxidant activity values of 5.23 mg TE/g using the DPPH method, and 3.92 mg TE/g using the ABTS method. Additionally, APN was found to contain total phenolic compounds and flavonoids at concentrations of 28.42 mg GAE/g and 19.63 mg CE/g, respectively (Figure 2b).

The HPLC analysis of APN phenolic compounds (Table 2) identified 17 distinct compounds. Chlorogenic acid was the most abundant, with a concentration of 8029.69 µg/g, while cinnamic acid was the least abundant, at 0.72 µg/g. The volatile compounds extracted from APN using SPME and identified by GC and GC-MS are shown in Table 3 with their aroma descriptions. Twenty-five volatile compounds were detected in the freeze-dried peel of APN. Ethyl acetate (fruity ester) was the most abundant compound at 13.25%, followed by hexanal (oily avocado) at 12.83%, while α-humulene (mild woody, earthy) was the least abundant at 0.12%. The fatty acid and phytosterol composition of APN is presented in Table 4. Oleic acid (C 18:1) was the predominant unsaturated fatty acid at 73.62%, followed by linolenic acid (C 18:3, ω 3) at 5.42%. The total unsaturated fatty acid content was 79.897%. Among saturated fatty acids, palmitic acid (C16:0) was the major component at 13.69%. The total phytosterol content in APN was 34.27%. Stigmasterol (16.13%) was the most abundant phytosterol, followed by campesterol (10.18%), while β-sitosterol (7.96%) was the least abundant. β-amyrine as a tritepenoid compound was present by 12.79%. The oxidizability (COX), atherogenicity index (AI), thrombogenicity index (TI), and the ratio of hypocholesterolemic to hypercholesterolemic (HH) fatty acids were 5.07, 0.171, 0.257, and 5.836, respectively (Table 4). These results indicate that APN may reduce the risk of cardiovascular diseases (CVDs).

### 3.2. Impact of Avocado Peel Nutraceutical on Paw Inflammation Volume

**Figure 3:**
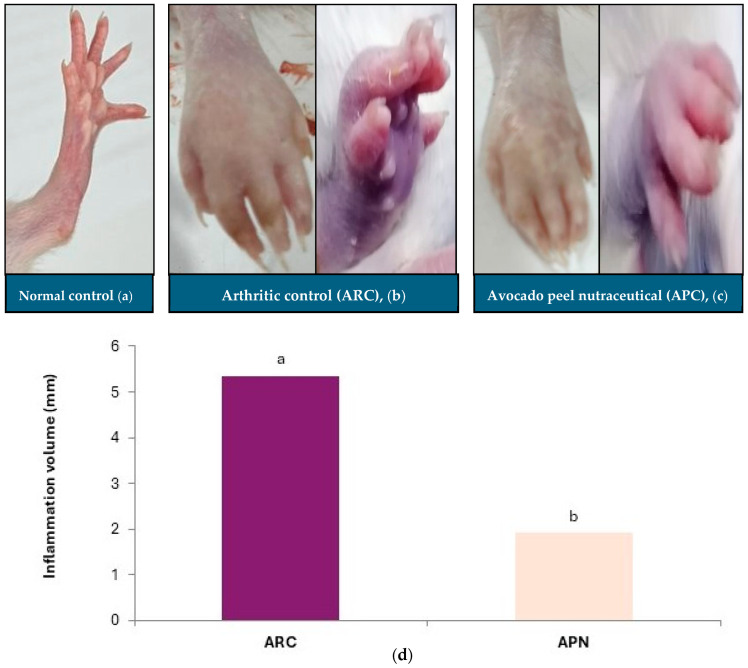
Effect of avocado peel nutraceutical on inflammation volume in arthritic rats. (**a**) Normal paw, (**b**) arthritic rat paw, (**c**) paw of rats treated with APN, (**d**) inflammation volume (mm) in experimental groups. Values are Mean ± SE, *n* = 6. Means with different letters are significantly different at *p* ≤ 0.05. ARC: arthritic control, APN: avocado peel nutraceutical.

Measuring paw swelling in rats is a straightforward, critical, and rapid method for assessing the degree of inflammation and evaluating the therapeutic efficacy of various treatments. Injection of FCA into the rat paw induced redness, swelling, and erythema compared to the non-injected paw (Figure 3). The paws of arthritic control rats exhibited a significant increase in inflammation, as indicated by the elevated paw volume (Figure 3b,d). Oral administration of APN to arthritic rats resulted in a significant reduction in paw inflammation volume (64%) compared to the arthritic control group (Figure 3c,d). The normal paw of the control group is shown in Figure 3a.

### 3.3. Impact of Avocado Peel Nutraceutical on Different Biochemical Parameters in Normal and Arthritic Rats

Table 5 summarizes the different biochemical parameters across all the studied groups. The injection of Freund’s complete adjuvant (FCA) as an arthritis inducer resulted in a significant increase in oxidative stress markers, as evidenced by a reduction in catalase (CAT) activity and an elevation in malondialdehye (MDA)—an indicator of lipid peroxidation—in the arthritic control group (Table 5). Inflammatory markers, including TNF-α, IL-1β, and IL-6 were significantly elevated in arthritic control rats compared to normal rats (Table 5). The plasma lipid profile of arthritic rats indicated significant dyslipidaemia, characterized by increased levels of total cholesterol (T-Ch), triglycerides (TG), and low-density lipoprotein cholesterol (LDL-Ch), along with a reduction in high-density lipoprotein cholesterol (HDL-Ch) (Table 5). Liver function markers (AST and ALT) and kidney function markers (creatinine and urea) were also significantly elevated in arthritic rats compared to normal rats (Table 5). Furthermore, lipid oxidation was confirmed in arthritic control rats by increased plasma levels of oxidized-LDL (Oxi-LDL) compared to normal rats. Administration of APN demonstrated significant improvements in oxidative stress, as shown by increased CAT activity and reduced MDA levels (Table 5). APN also exhibited anti-inflammatory activity, significantly reducing the levels of inflammatory markers (TNF-α, IL-1β and IL-6) to varying degrees compared to the arthritic control group. Additionally, the impaired liver (AST and ALT) and kidney (creatinine and urea) functions observed in arthritic control rats were improved in rats administered with APN. Rats treated with APN showed significant improvements in dyslipidaemia, as indicated by reduced levels of T-Ch, TG, and LDL-Ch, along with a substantial increase in HDL-Ch. Oxi-LDL levels also decreased significantly in APN-treated rats compared to the arthritic control group (Table 5).

The results of nutritional parameters (Table 5) revealed that body weight gain and final body weight were significantly reduced in the arthritic control group compared to other groups, while spleen weight (%) increased significantly in the same group. Administration of APN led to improvements in all studied nutritional parameters compared to the arthritic control group.

### 3.4. Impact of Avocado Peel Nutraceutical on the Coronary Risk Index and Atherogenic Index

**Figure 4:**
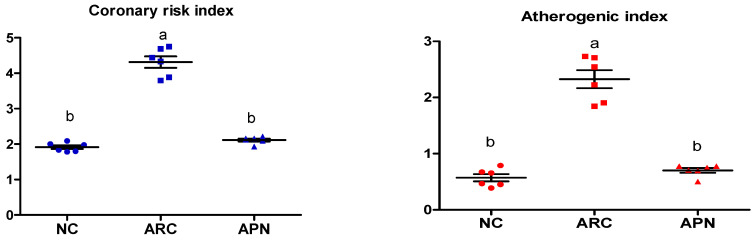
Effect of APN on coronary risk index and atherogenic index in arthritic rats. Coronary risk index = T-Ch/HDL-Ch, atherogenic index = LDL-Ch/HDL-Ch. Values are mean ± SE, *n* = 6, means with different letters show significant difference between values at probability level of 0.05. NC: normal control; ARC: arthritic control; APN: avocado peel nutraceutical.

The coronary risk index and atherogenic index were significantly (*p* ≤ 0.001) elevated in arthritic control rats in comparison to normal rats, as shown by increased plasma levels of T-Ch/HDL-Ch and LDL-Ch/HDL-Ch, respectively (Figure 4) APN administration led to a significant (*p* ≤ 0.001) reduction in both indices (Figure 4).

### 3.5. Impact of APN on the Expression of COX-2, IL-6, IL-1β, IL-10, and TNF-α in Different Rat Groups

**Figure 5:**
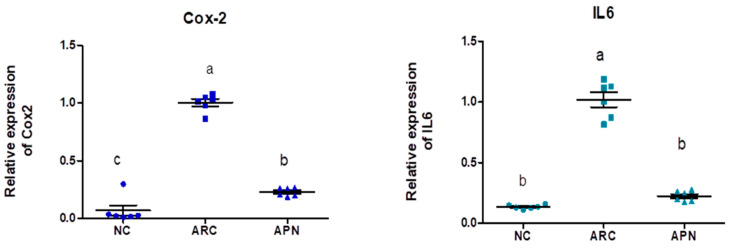

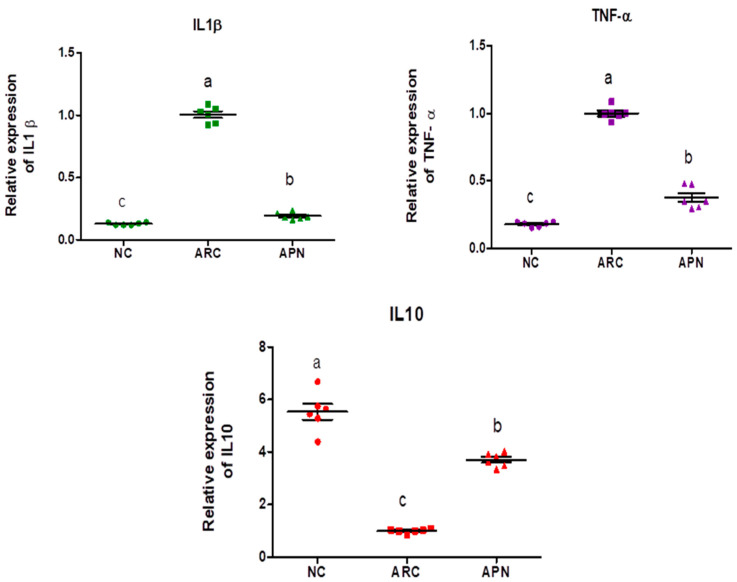
The mRNA expression COX-2, IL-6, IL-1β, IL-10, and TNF-α in the liver of different experimental groups. The mRNA expression COX-2, IL6, IL-1β, IL-10, and TNF-α is normalized with housekeeping gene (GAPDH). Values are represented as means ± SE, different letters show significant difference between values at a probability level of 0.05.

**Figure 6:**
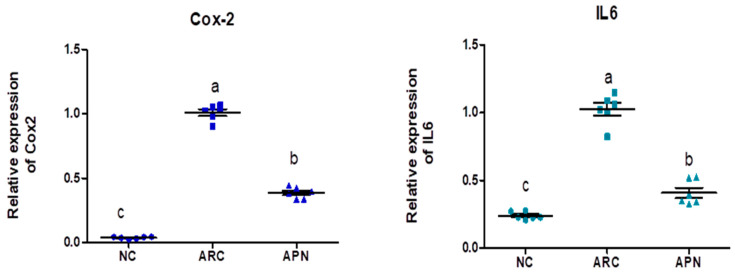

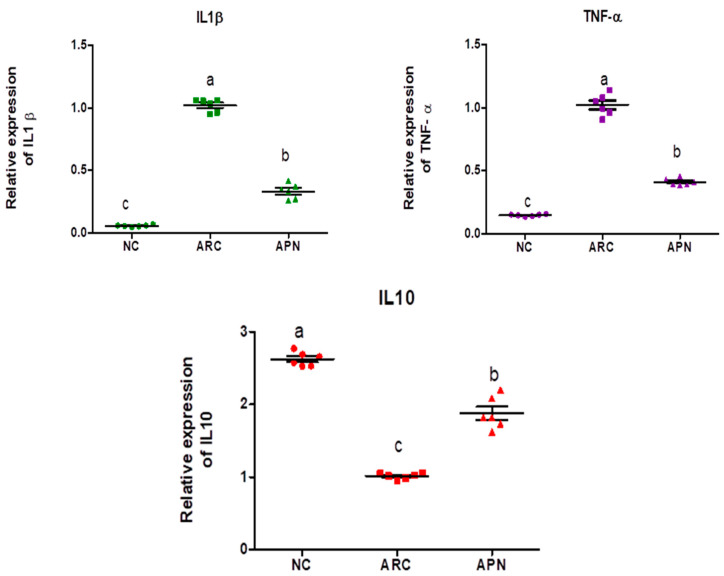
The mRNA expression COX-2, IL-6, IL-1β, IL-10, and TNF-α in the spleen of different experimental groups. The mRNA expression COX-2, IL6, IL1β, IL10, and TNF-α is normalized with a housekeeping gene (GAPDH). Values are represented as means ± SE, different letters show significant difference between values at a probability level of 0.05.

The expression of COX-2, IL-6, IL-1β, IL-10, and TNF-α genes was assayed by RT-PCR in the liver and spleen tissues (Figure 5 and Figure 6). COX-2, IL-6, IL-1β, and TNF-α gene expression were significantly upregulated in the liver and spleen of the arthritic control group compared to the normal control group. IL-10 gene expression was significantly downregulated in the liver and spleen of arthritic rats. APN treatment significantly (*p* < 0.001) downregulated the expression of COX-2, IL-6, IL-1β, and TNF-α genes in the liver when compared to the arthritic control group by 4.4-, 4.6-, 5.2-, and 2.7-fold change, respectively. Similarly, APN treatment significantly (*p* < 0.001) downregulated the expression of COX-2, IL-6, IL-1β, and TNF-α genes in the spleen in comparison to the arthritic control group by 2.6-, 2.5-, 3.1-, and 2.5-fold change, respectively. The mRNA expression of IL10 gene was significantly (*p* < 0.001) upregulated by APN treatment by 3.7- and 1.9-fold change in the liver and spleen, respectively.

### 3.6. 16S rRNA Amplicon Sequencing Analysis of Colon Content

#### 3.6.1. Diversity and Richness Analysis

**Figure 7:**
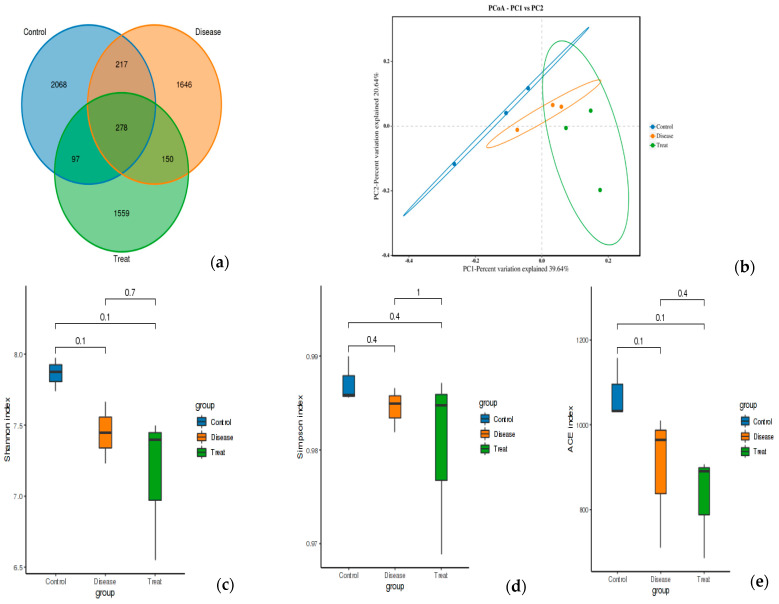
Feature analysis, Alpha diversity, and PCoA analysis of gut bacteria in different experimental groups. (**a**) Venn diagram, (**b**) PCOA, (**c**) Shannon index, (**d**) Simpson index, (**e**) ACE index. Control = normal control; Disease = ARC group (arthritic control group); Treat = APN group.

The effect of RA on the diversity of the gut microbiota and the potential modulation with APN was tested using 16S rRNA metagenomic sequencing of colon content (Figure 7). Common and unique features among groups were visualized using Venn diagrams (Figure 7a). The findings of Alpha diversity showed higher diversity and richness in the control group compared to the other two groups with non-significant differences in either diversity or richness within all three test groups using ACE, Shannon, and Simpson indices (Figure 7c–e). PCoA analysis showed distinct clusters for each test group (Figure 7b).

#### 3.6.2. Relative Abundance

**Figure 8:**
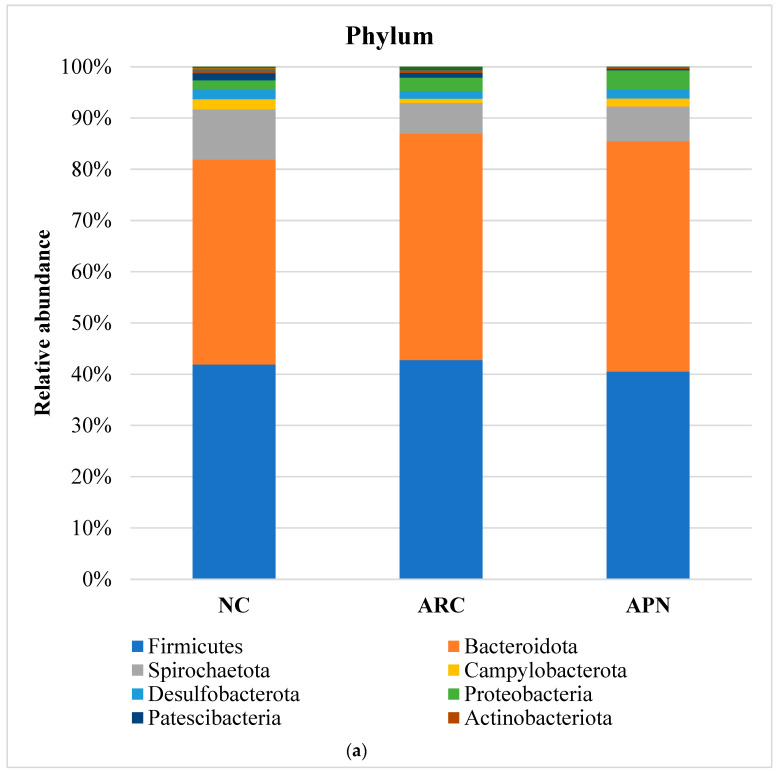

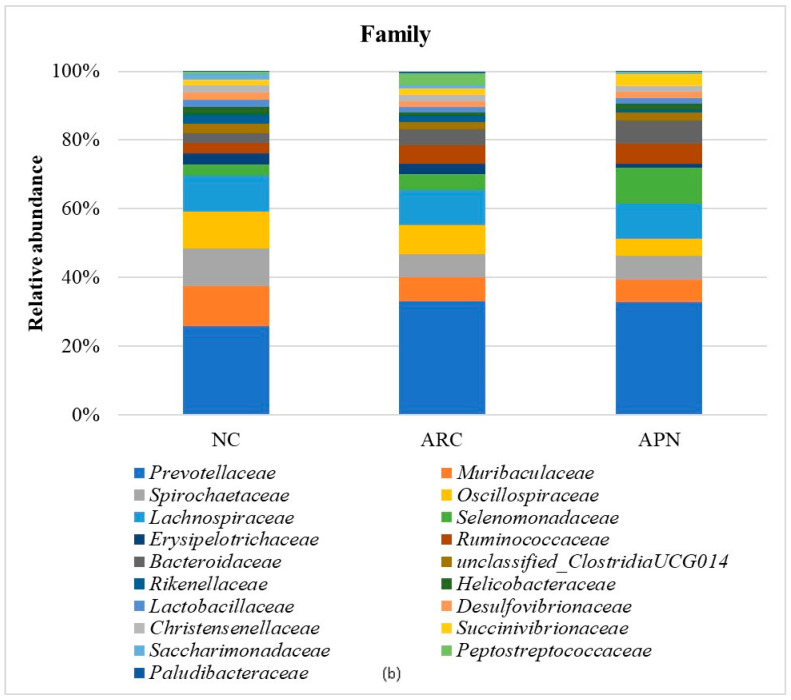

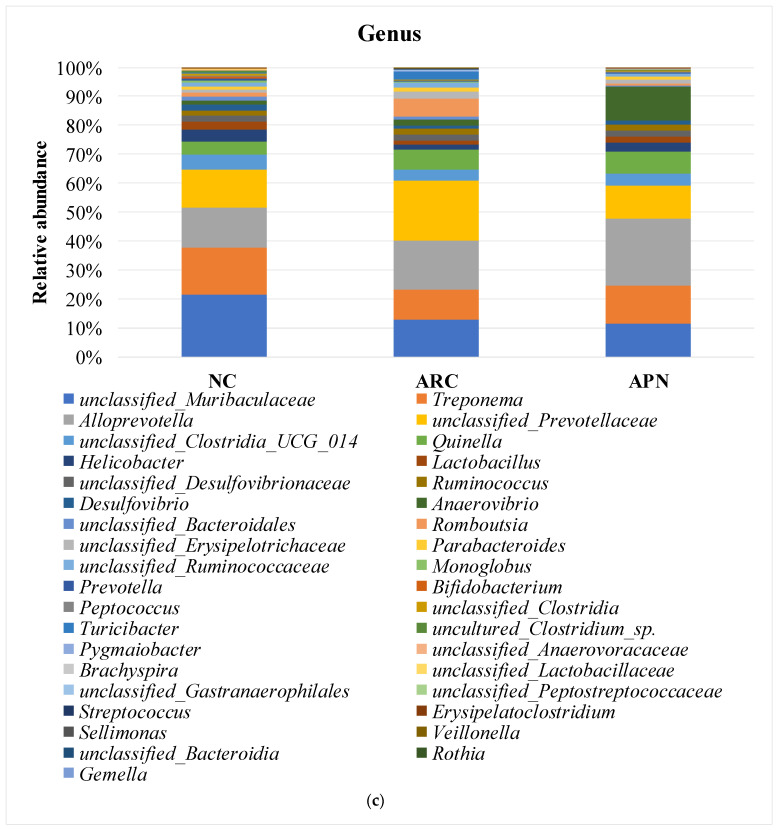

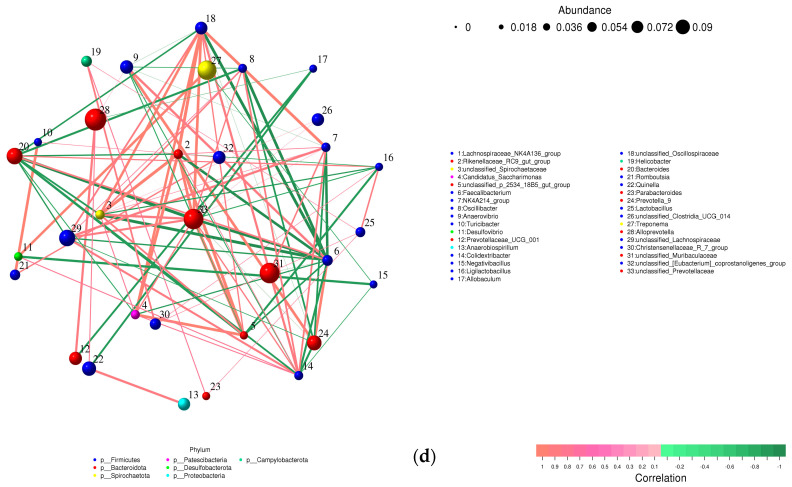
Relative abundance of bacterial phyla, family, and genus in colon content samples of different experimental groups and the correlation network of the different bacterial community interactions. (**a**) Phyla, (**b**) family, (**c**) genus, (**d**) correlation network (color gradient indicates Spearman’s correlation coefficients).

The top five most abundant phyla were Firmicutes, Bacteroidota, Spirochaetota, Campylobacterota, and Desulfobacterota (Figure 8a). Although the relative abundance of these phyla showed subtle differences between the three test groups, none of the differences were found to be significant. However, it is noteworthy that the abundance of Firmicutes and Cyanobacteria phyla in the arthritic control group was elevated compared to the normal control group. This elevation was reversed in the rats treated with APN. Additionally, treatment with APN increased the abundance of Spirochaetota, Campylobacterota, and Desulfobacterota (Figure 8a).

At the family level (Figure 8b), *Prevotellaceae*, *Muribaculaceae*, *Spirochaetaceae*, *Oscillospiraceae*, and *Lachnospiraceae* were the dominant taxa across all test groups. Although no significant differences were observed between groups after adjusting the *p* value, a notable decline in *Muribaculaceae* and *Monoglobaceae* (*p* values ≤ 0.05) was observed in arthritic rats compared to the normal control group. The abundance of *Peptostreptococcaceae* increased 4.68-fold in the ARC group compared to the normal control group. However, this increase was reduced 8.48-fold following treatment with APN.

Comparable findings were seen at the genus level with a few early signs of dysbiosis in the arthritic group compared to normal rats (Figure 8c). This was seen in the decline in abundances of *Lactobacillus*, *unclassified_Lactobacillaceae*, *Bifidobacterium*, *Monoglobus* (*p* = 0.0354) *unclassified_Muribaculaceae* (*p* = 0.0394), *unclassified_Clostridia* (*p* = 0.0589), and *uncultured_Clostridium_sp*. (*p* = 0.0158). The same group showed a clear enrichment in the abundances of *Turicibacter*, *Romboutsia*, *unclassified_Prevotellaceae*, and *unclassified_Peptostreptococcaceae.* Although non-significant, a restorative effect was detected upon treatment with APN in some genera (e.g., *Lactobacillus*, *unclassified_Lactobacillaceae*, *Turicibacter*, *Romboutsia*, and *unclassified_Prevotellaceae*), showing abundance levels similar to normal rats.

A correlation network was created to illustrate the different bacterial community interactions (Figure 8d). *Turicibacter* had a significant positive correlation with *Romboutsia* (r = 0.9910, *p* ≤ 0.0001). There was a significant positive correlation between *Prevotellaceae* and *Alloprevotella* or *Quinella* (r = 0.7674, *p* = 0.0158; r = 0.7336, *p* = 0.0245). *Prevotella* was positively correlated with *Anaerovibrio* (r = 0.9074, *p* = 0.0007). *Allobaculum* had a significant positive correlation with *unclassified_Muribaculaceae* (r = 0.7140, *p* = 0.0307). On the other hand, a significant negative correlation was observed between *Lactobacillus* and *Alloprevotella* (r = −0.6786, *p* = 0.044).

**Figure 9:**
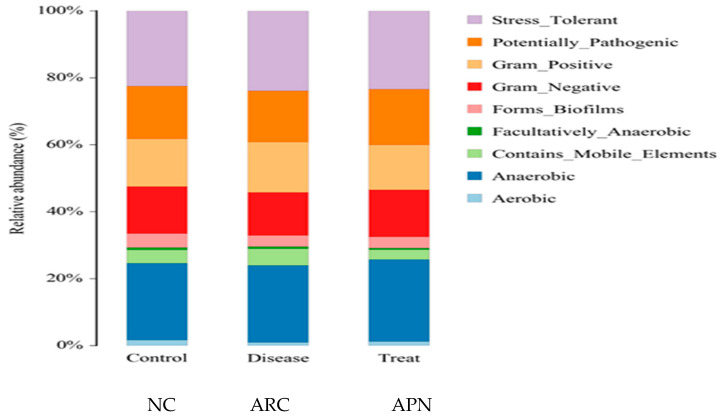
Prediction of high-level phenotypes in different groups using the BugBase function prediction algorithm.

Figure 9 shows the functional prediction of the top phenotypes across different test groups using the BugBase algorithm. Changes in bacterial phenotypes provide insights into disease progression and prognosis. Only subtle changes in traits were observed in arthritic rats compared to the normal control group. Stress-tolerant taxa within the gut bacterial community were enriched, along with those harboring mobile genetic elements associated with virulence. In contrast, a reduced abundance of aerobic and gram-negative bacteria was detected in arthritic rats compared to the normal control group.

### 3.7. Molecular Docking Studies of Avocado Peel Nutraceutical

The results of the binding affinity of APN phytochemicals with protein tyrosine phosphatase non-receptor type 22 (PTPN22) are shown in Table 6. The results showed that the phenolic compounds, volatile compounds, fatty acids, and phytosterols exhibited different binding interactions with PTPN22. Among all the tested compounds, β-amyrinephytosterol and ellagic acid phenolic compound showed the highest binding affinity for PTPN22. Ligands such as Oleic acid, Linolenic acid, and Linoleic acid (fatty acids) showed moderate binding affinities. In contrast, Hexanal, Ethyl acetate, Methyl propanoate, and Methional (volatile compounds) exhibited the lowest binding affinity.

The 3-D and 2-D structures of the compounds exhibiting the highest binding affinities to PTPN22are shown in Table 7. Ellagic acid, quercetin, naringenin, β-amyrine, campesterol, β-sitosterol, and Stigmasterol interacted with the active site of PTPN22 at varying numbers of amino acid residues with different types of bonds. Ellagic acid interacted with the active site of PTPN22 at the TYR44, SER271, TYR66, LYS39, and PRO270 residues. Naringenin interacted with the active site of PTPN22 at the TYR44, TYR38, TYR66, LYS61, LYS39, and LEU6 residues with conventional hydrogen bonds, Pi-Pi T-shaped, and Pi-Alkyl bonds. Quercetin interacted with the active site of PTPN22 at the THR46, TYR66, TYR38, LYS39, LYS42, and PRO45 residues with conventional hydrogen bonds, Pi-Sigma, Pi-Pi T-shaped, and Pi-Alkyl bonds. Hydrogen bonds and hydrophobic interactions formed between β-amyrine and the LEU261, SER257, ILE251, LEU246, PRO253, TYR94, TYR242, and PHE256 residues of PTPN22. β-Sitosterol and campesterol interacted with the active site of PTPN22 at the SER271, LYS39, PRO45, PRO270, LYS42, and TYR66 residues with conventional hydrogen bonds, Alkyl, and Pi-Alkyl bonds. Hydrophobic interactions such as Alkyl and Pi-Alkyl interactions formed between Stigmasterol and the LYS39, ILE63, PRO270, LYS32, and TYR66 residues of PTPN22.

## 4. Discussion

It is well established that Freund’s complete adjuvant (FCA) induces an arthritis model in rats that closely mimics rheumatoid arthritis (RA) in humans, as supported by extensive prior research [38,48,49]. FCA induces arthritis in rats through a strong inflammation response, induction of immune response, and oxidative stress. It was previously reported that oxidative stress and inflammation play an important role in rheumatoid arthritis both in experimental animals and clinical studies [49,50,51,52].

Inflammatory cytokines play a crucial role in the host defense mechanism [53]. The increase in inflammatory cytokines is associated with joint inflammation, damage, and hyperplasia of synovial fluids [54] as observed in the current study through redness, swelling, and elevation of paw volume in arthritic rats. Inflammatory cytokines, TNF-α, IL-1β, and IL-6, are necessary in triggering joint damage and inflammation [55]. The increase in inflammatory cytokines leads to cachexia [38,56] as observed in the present study by a non-significant reduction in the final body weight of arthritic rats. The inflammatory cytokine TNF-α induces and regulates other cytokines such as IL-6 and IL-1β [57,58]. TNF-α, IL-1β, and IL-6, were significantly increased in plasma, in association with upregulation in the expression of inflammatory marker genes (COX2, TNF-α, IL-1β and IL-6) and downregulation of the anti-inflammatory cytokine IL-10 in liver and spleen tissues of arthritic rats. The increase in inflammatory cytokines in plasma (IL-6, TNF-α, and IL-1β) and in liver and spleen tissues (COX2, TNF-α, IL-1β and IL-6) observed in the present study confirms the results of Laragione et al. [54]. The downregulation of IL-10 observed in the current study is a valuable indicator of the disturbances in immune system and immune response of arthritic rats. IL-10 is a vital anti-inflammatory cytokine that acts as a negative regulator of immune responses by limiting immune cell activation in the innate immunity, thereby reducing inflammatory cytokines [51,59]. Consequently, IL-10 manages autoimmune diseases, such as rheumatoid arthritis, by suppressing the immune response, which prevents tissue damage caused by uncontrolled inflammatory responses [60]. Additionally, disturbances in the immune system were observed in arthritic rats through an elevation of spleen weight, and these results are supported by the findings of Hassan et al. [38]. The spleen is a vital organ for immune response and regulation, functioning to generate immune cells and filter dead cells. The increase in the spleen index in arthritic rats may be explained by immune cell filtration as well as the accumulation of dead WBCs and RBCs [61,62].

In the present study, cardiovascular risk was observed in arthritic rats via dyslipidaemia, elevation of oxidized-LDL, and an increase in the incidence of the coronary risk index (T-Ch/HDL-Ch) and atherogenic index (LDL-Ch/HDL-Ch). Rheumatoid arthritis is an inflammatory autoimmune disease that primarily affects the joints [4]. Cardiovascular disease is a leading cause of mortality in RA patients [6]. The cardiovascular manifestations in RA include atherosclerosis, heart failure, and myocarditis. Inflammation is a principal mediator of endothelial dysfunction and a crucial driver of cardiovascular risk and complications in patients with RA [63]. Dyslipidemia is commonly observed in rheumatoid arthritis patients, with a prevalence ranging between 55% and 65% [64]. Inflammatory cytokines—such as TNF-α and IL-6—produced in the local joint can enter the circulation and lead to systemic inflammation and lipid metabolism abnormalities [64]. Elevation of IL-6 is a key factor in the incidence of cardiovascular diseases in RA patients [65]. In the current study, the elevation of oxidized-LDL levels in plasma is one of the main causes of the high cardiovascular risk observed in arthritic rats. It was previously reported that lipid peroxidation induces oxidative modification of lipids in different cells and membranes, leading to the formation of oxidized-LDL [66].

Administration of avocado peel nutraceutical in arthritic rats improved oxidative stress, associated with a decrease in inflammatory markers in plasma and their gene expression in liver and spleen tissues, while the expression of the anti-inflammatory cytokine IL-10 was upregulated. In addition, APN demonstrated cacceptardioprotective activity against the incidence of cardiovascular diseases in arthritic rats by improving dyslipidaemia and reducing coronary risk index and atherogenic index. These results suggest that inhibiting inflammation and oxidative stress may be the underlying mechanisms by which APN alleviates arthritis. The improvement in various biochemical parameters observed in the present study may be attributed to the presence of different phytochemicals identified in APN.

In the present study, oxidative stress was reduced in arthritic rats that administered APN. APN contains various phytochemicals, such as phenolic compounds and phytosterols, which possess antioxidant activity in vitro (DPPH and ABTS assays)—as observed in the current study—and in vivo through the reduction of oxidative stress and MDA levels, along with an elevation in catalase activity in association with a reduction in oxidized-LDL in rats fed APN for 21 days. The results revealed that APN contains chlorogenic acid as a major phenolic compound. Previous studies also reported the presence of chlorogenic acid in avocado peel [67,68]. Chlorogenic acid exhibits various biological activities, including anti-inflammatory, antioxidant, and cardioprotective effects [20,69,70]. The phytosterols present in APN demonstrate antioxidant, anti-inflammatory, anti-arthritic, and cardioprotective activities [71,72,73]. Thus, APN may be considered a source of phytochemicals with diverse biological activities.

The reduction in cardiovascular disease risk observed in rats administered with APN orally may be attributed to the presence of oleic acid and different phytosterols in the nutraceutical, as demonstrated by the phytochemical screening of APN. Avocado fruit and its by-products are rich sources of phytosterols, particularlyβ-sitosterol, and stigmasterol [21,74,75,76]. Phytosterols exhibit cardioprotective effects by inhibiting cholesterol absorption and reducing cholesterol synthesis in the liver [29]. Previous studies have demonstrated that oleic acid and monounsaturated fatty acid lowers the risk of cardiovascular diseases [77] by improving dyslipidemia and reducing inflammation, as shown in the current results. Additionally, oleic acid has demonstrated anti-inflammatory effects by reducing the expression of inflammatory marker genes, such as TNF-α and IL-6 [78] as observed in our results. The upregulation of IL-10 gene expression observed in rats administered with APN may reduce the cardiovascular risk by downregulating COX2 expression. IL-10 plays a vital role in inhibiting COX-2 expression, which contributes to plaque formation [64], thereby reducing the incidence of CVDs. The low values of AI (0.171) and TI (0.229), along with the high H/H ratio (5.836) observed in the current study, indicate that APN can be considered a cardioprotective nutraceutical capable of mitigating cardiovascular risk in arthritic rats. The incidence of CVDs increases in rheumatoid arthritis patients [6]. Thus, APN prepared in the present study may protect rheumatoid arthritis patients from CVDs. These findings support a study by Marović et al. [79], who found that avocado peel exhibited an H/H ratio of 3.55, indicating significant hypocholesterolemic potential. The AI and TI indices characterize the impact of individual fatty acids oils on human health, particularly the development of atherosclerosis and the incidence of atheroma, blood clots, and thrombi [80,81].

Growing evidence links alterations in the gut bacterial community to the incidence and progression of autoimmune diseases [8,82]. Recent efforts have been made to conduct more in-depth studies exploring the importance of the gut–joint axis in the development of RA to confirm the causality claim for gut microbiota perturbation [83,84]. The impact of specific gut bacterial taxa on immune function through metabolite production and upregulation of immune cells has been demonstrated in both animal models and clinical studies [15,16,84,85]. Loss of eubiosis in the gut can mediate the pathogenesis of RA through metabolic pathways involving the recruitment of lymphocytes (Th17, Tregs), cytokine production, and altered short-chain fatty acid (SCFA) production. Such pathways have been found to involve gut bacteria of specific lineages [86,87].

Our study revealed clear signs of dysbiosis at different taxonomic depths. The subtle increase in Firmicutes and Cyanobacteria in arthritic rats might be due to the high diversity of these phyla, which include many pathogenic and toxin-producing species capable of triggering adverse immune responses. Lee et al. [88] reported a lower ratio of Bacteroidota/Firmicutes in RA patients. Cyanobacteria can be a source of lipopolysaccharide (LPS), which is linked to the loss of intestinal tight junctions, chronic inflammation, aberrant innate immune responses, and cardiovascular disease [89,90]. The decline in some metabolically beneficial and SCFA-producing taxa in the ARC group (e.g., *Muribaculaceae*, *Monoglobaceae*, *unclassified_Lactobacillaceae*, *Monoglobus Lactobacillus*, and *Bifidobacterium*) supports the growing evidence linking RA to altered metabolic patterns via the gut–joint axis [91,92]. The same can be inferred from the lower abundances of specific members of the *Clostridia* family in the ARC group. *Clostridium* bacteria are producers of butyrate, which contributes to maintaining intestinal tight junctions and reducing inflammation [93].

*Turicibacter* has been previously linked to adverse immune responses and overexpression of lymphocytes [94]. It has also been associated with cardiovascular disease through modulation of lipid composition and bile acid production [95,96]. *Romboutsia* is commonly associated with *Turicibacter* in arthritic subjects [97]. The present study supports these findings with a positive correlation and remarkable blooming of both genera in the ARC group.

Treatment with APN in this study presents a new approach with promising early signs of restoring gut homeostasis in RA. This may be due to the plant’s rich content of phenolic compounds. These functional food components have a long record of modulating gut ecology through different mechanisms. Several reports have demonstrated their corrective impact on immune function, intestinal health, and metabolism [98,99,100]. This was reflected in our study through the modulation of key pathobiontic bacterial taxa (Firmicutes, Cyanobacteria, *Turicibacter*, *Romboutsia*, and *unclassified_Prevotellaceae*). More evidence is seen in the enrichment of *Lactobacillus* and *unclassified_Lactobacillaceae* to near-normal levels.

The cardiovascular risk associated with RA can be attributed to the involvement of gut bacterial metabolites in the progression of cardiovascular disease. Bile acids, SCFAs and LPS are common byproducts of bacterial metabolism of dietary components in the gut. Metabolites from specific species have been reported to mediate cardiometabolic disease [3,101]. In this study, the risk of cardiovascular adverse events in RA is shown in the enriched abundance of *Turicibacter* in arthritic rats compared to normal rats. This can also be noted in the emergence of the *Gemella* genus in the arthritic group alone. According to recent reports, this genus has been implicated in coronary heart disease and endocarditis [102]. The cardiovascular risk can also be attributed to other metabolic changes involving the gut ecology during the progression of RA, such as elevated lipid profiles, low-grade inflammation, and oxidative stress [103].

Two interesting findings in this study were the unchanged abundances of *Prevotella* and *Helicobacter* genera in arthritic rats. A review by Precup and Vodnar in 2019 explains the wide genetic diversity within and between species of the *Prevotella* genus. Only selected species exhibit pathobiontic behavior, while others demonstrate metabolic benefits. Some studies have mentioned the involvement of *Helicobacter* in a gut–joint-axis-mediated RA. However, not all studies support this claim. A clinical study involving 56,000 *H. pylori*-infected RA patients found no association between *Helicobacter* and RA [104].

In the current research, rats were administered orally with APN. Oral delivery is the most common and acceptable administration for patients and thus has a high market share. However, most oral drugs show low bioavailability due to their low solubility in gastrointestinal fluids, degradation by strong acids and enzymes in the GI tract, poor absorption by intestinal epithelial cells, and first-pass hepatic metabolism [105]. In the present research, APN non-polar compounds increase the absorption of polar molecules in the gastrointestinal tract and enhance their bioavailability and absorption. Additionally, APN contains oleic acid (C18:1) and long chain fatty acids, which have been shown to increase the permeability of a series of hydrophilic compounds by dilating the tight junction and/or changing the cytoskeleton of the intestinal epithelial cells. One of the foremost advantages of these excipients is the comfort of incorporating into the conventional oral dosage forms without the need for complex or expensive formulation techniques [106]. In the present study, the polar compounds present in the studied APN protect non-polar bioactive compounds (e.g., fatty acids) from oxidation by free radicals present in the body and produced during metabolism. The incorporation of bioactive fatty acid sources in nutraceutical/dietary supplements faces several challenges due to the high susceptibility of fatty acids to oxidation, which causes rancidity, consumer rejection, and a decrease in nutritional value [107,108]. Thus, both polar and non-polar compounds present in the APN synergize their activities and bioavailability during the treatment of arthritic rats.

Several in silico studies provide insights into the potential of new active compounds and therapeutic targets for the treatment of RA. Deng et al. [109] showed that β-sitosterol, stigmasterol, kaempferol, quercetin, and 2-methoxy-3-methyl-9,10-anthraquinone have strong binding affinity with proinflammatory cytokines (TNF-α, IL-6, IL-10), mitogen-activated protein kinase1, transcription factor p65, and RAC-α serine/threonine-protein kinase—all of which are RA-related hub targets. Another molecular docking study [110] identified novel compounds with similar structure to phytochemicals in *Embeli aribes* (embelin, embelinol, rapanone, catechin, and β-sitosterol) that showed promising binding affinity towards target proteins involved in RA pathophysiology (Bruton’s tyrosine kinase protein, p38 mitogen-activated protein kinases, interleukin-1 receptor-associated kinase 4, and matrix metallopeptidase 9). The active ingredients in *Sinomenium acutum* (beta-sitosterol, 16-epi-Isositsirikine, sinomenine, and stepholidine) have potential interactions with target proteins (caspase-3, prostaglandin-endoperoxide synthase 2, and transcription factor Jun) involved in the treatment of RA [111]. Protein tyrosine phosphatase non-receptor type 22 (PTPN22) gene is a major genetic risk factor for a number of autoimmune diseases. PTPN22 is a gene encoding a protein involved in the regulation of immune cell signaling. Polymorphisms in PTPN22 are associated with the incidence of rheumatoid arthritis [112], leading to immune dysregulation through the stimulation of the immune system’s main components and osteoclasts [113]. The PTPN22 protein is negatively regulated by T-cell receptor signaling. Dysregulation of PTPN22 increases the percentage of CD4+ T cells, leading to higher production of proinflammatory cytokines and inflammatory responses in RA [114]. PTPN22 regulates NLRP3 inflammasome activity by modulating upstream signaling pathways, particularly Toll-like receptors and NF-κB signaling. Dysregulation of PTPN22 contributes to excessive NLRP3 activation, promoting the production of proinflammatory cytokines like IL-1β and IL-18, which exacerbate inflammatory responses [115]. IL-1 β increases cytokines and chemokines secretion, promotes Th17 cell differentiation, and reduces the synthesis of cartilage components [116]. Recent reports have mentioned that PTPN22 ablation accelerates arterial thrombus formation and rat tail-bleeding time, suggesting that PTPN22 is a potential therapeutic target for cardiovascular diseases, atherosclerosis, and calcific aortic valve disease [11,117,118,119]. As cardiovascular diseases are the most common complications in RA patients, the inhibition of PTPN22 represents a potential therapeutic target for autoimmune diseases such as RA and its complications. In the present study, molecular docking analysis of phytochemicals in APN revealed varying binding affinities with PTPN22. Phenolic compounds (ellagic acid, quercetin, naringenin) and phytosterols (β-amyrine, campesterol, β-sitosterol, stigmasterol) showed the highest binding affinity with PTPN22, suggesting their potential as PTPN22 inhibitors.

This study had two main limitations: small sample sizes and a relatively short experimental duration. As this is the first report on the effects of avocado peel on RA, and given that the diversity and composition of the gut microbiota have been correlated with the duration of RA disease, further validation is required using different experimental models with larger sample sizes and longer durations (8–12 weeks). Additionally, integrated multi-omics (e.g., metabolomics and metagenomics) are recommended in future studies for more comprehensive understanding.

## 5. Conclusions

The induction of RA led to inflammatory and immune response, along with early signs of dysbiosis, in arthritic rats. Bacterial taxa associated with inflammation, impaired immune function, and cardiometabolic diseases were enriched in the ARC group. APN demonstrated promising anti-arthritic activity and ameliorated the risk of cardiovascular diseases by reducing inflammation and oxidative stress, as well as regulating the expression of genes related to inflammatory and anti-inflammatory cytokines. Treatment with APN also showed potential in restoring eubiosis in the gut through the modulation of specific RA-associated taxa. Nutraceuticals derived from edible plant sources may represent a promising approach to combating autoimmune diseases and their associated cardiovascular risks.

## Figures and Tables

**Table 1:** Primers pair sequences used for real-time PCR amplifications.

Target Genes	Sequence	Ref
COX-2	FW (5′-TGGTGCCGGGTCTGATGATG-3′) RW (5′-GCAATGCGGTTCTGATACTG-3′)	[42]
IL6	FW (5′-ACTGAACTTCGGGGTGATTG-3′) RW (5′-GCTTGGTGGTTTGCTACGAC-3′)	[43]
IL-1β	FW (5′-TGA TGG ATG CTT CCA AAC TG-3′) RW (5′-GAG CAT TGG AAG TTG GGG TA-3′)	[43]
IL10	FW (5′-CTTACTGGCTGGAGTGA-3′) RW (5′-AGCTCTCGGAGCATGTG-3′)	This study
GAPDH	FW (5′-GTATTGGGCGCCTGGTCACC-3′) RW (5′-CGCTCCTGGAAGATGGTGATGG-3′)	[43]

**Table 2:** HPLC of avocado peel nutraceutical phenolic compounds profile (µg/g).

Phenolic Compounds	APN(µg/g)
Gallic acid	274.41 ± 0.076
Chlorogenic acid	8029.69 ± 0.067
Methyl gallate	200.84 ± 0.086
Coffeic acid	76.43 ± 0.054
Syringic acid	31.44 ± 0.066
Pyro catechol	235.61 ± 0.045
Ellagic acid	112.89 ± 0.095
Coumaric acid	92.23 ± 0.076
Vanillin	4.23 ± 0.052
Ferulic acid	13.54 ± 0.015
Naringenin	165.82 ± 0.041
Rosmarinic acid	49.35 ± 0.051
Daidzein	5.30 ± 0.0105
Querectin	66.70 ± 0.056
Cinnamic acid	0.72 ± 0.040
Kaempferol	14.32 ± 0.065
Hesperetin	14.29 ± 0.046

**Table 3:** Volatile compounds of avocado peel nutraceutical.

Volatile Compounds	LRI ^a^	Peel ^b^	Aroma Description ^c^
Ethyl ethanoate	601	1.59 ± 0.001	
Ethanol	617	0.57 ± 0.001	Herbaceous, fruity
Ethyl acetate	648	13.25 ± 0.001	Fruity ester
Methyl propanoate	657	1.46 ± 0.001	
Butanal	675	11.08 ± 0.002	Malty, green odor
Propyl acetate	701	2.16 ± 0.001	
Methyl butyrate	726	1.34 ± 0.001	
1-Penten-3-ol	731	0.08 ± 0.001	Fresh, mild fruity
(E)-2-Hexenal	775	3.79 ± 0.001	Green, fruity
Pentanol	778	0.07 ± 0.001	Fresh, mild fruity
Hexanal	803	12.83 ± 0.002	Oily avocado
2-Furfural	835	8.27 ± 0.001	Sweet, coffee
Isobutyl propanoate	865	4.37 ± 0.001	
Isoamyl acetate	874	2.38 ± 0.001	
Methional	896	6.07 ± 0.001	
Benzaldehyde	984	8.26 ± 0.001	
Ethyl hexanoate	1019	4.67 ± 0.001	
γ-Terpinene	1067	2.06 ± 0.001	
(Z)-7-Decenol	1196	0.06 ± 0.001	
Decanal	1217	9.52 ± 0.001	Orange peel
(E,Z)-2,4-Decadienal	1296	3.56 ± 0.001	
α-Humulene	1459	0.12 ± 0.001	Mild woody, earthy
(E,E) α-Farnesene	1512	0.29 ± 0.001	
δ-Cadinene	1526	1.03 ± 0.001	
Z-Nerolidol	1539	0.08 ± 0.001	

^a^: LRI: linear retention index; ^b^: values are expressed as area percentages. ^c^: Aroma description cited from Kebede et al. and Komthong et al. [46,47].

**Table 4:** Fatty acids and phytosterols content of avocado peel nutraceutical.

Parameters	APN
Fatty acids (as a percentage of total fatty acids)	
Palmitic acid (C16:0)	13.69 ± 0.055
Oleic acid (C18:1)	73.62 ± 0.064
Linoleic acid (C18:2) (ω − 6)	0.857 ± 0.004
Linolenic acid (C18:3) (ω − 3)	5.42 ± 0.066
Total saturated fatty acids	**13.69 ± 0.055**
Total unsaturated fatty acids	**79.897 ± 0.011**
Ratio of ω − 3:ω − 6	**6.3 ± 0.002**
COX = (C18: 1 + 10.3 × C18: 2 + 21.6 × C18: 3)/100	**5.07 ± 0.001**
AI = (C12: 0 + 4 × C14: 0 + C16: 0)/(ΣMUFA + Σ(ω − 3) + Σ(ω − 6))	**0.171 ± 0.001**
TI = (C14: 0 + C16: 0 + C18: 0)/(0.5 × MUFA) + (0.5 × Σ(ω − 6) + (3 × Σ(ω − 3)) + (ω − 3/ω − 6))	**0.229 ± 0.001**
H/H ratio = (C18:1 + C18:2 + C18:3)/(C16:0)	**5.836 ± 0.002**
Triterpenoids and Phytosterols (as a percentage of total unsaponifiable matter)	
β-Sitosterol	7.96 ± 0.007
Stigmasterol	16.13 ± 0.01
Campesterol	10.18 ± 0.008
β-amyrine	12.79 ± 0.011
Total phytosterols	**34.27 ± 0.011**

**Table 5:** Biochemical and nutritional parameters of different studied groups.

Parameters	NC	ARC	APN
Oxidative stress status			
MDA (nmol/mL)	7.85 ^c^ ± 0.34	19.00 ^a^ ± 0.97	11.83 ^b^ ± 0.60
CAT (U/L)	614.17 ^a^ ± 8.98	304.17 ^c^ ± 7.57	519.17 ^b^ ± 7.57
Inflammatory markers			
TNF-α (pg/mL)	15.5 ^c^ ± 0.43	33.17 ^a^ ± 0.91	20.50 ^b^ ± 0.76
IL-1β (pg/mL)	39.14 ^b^ ± 0.35	42.75 ^a^ ± 0.99	36.13 ^c^ ± 0.39
IL-6 (pg/mL)	62.42 ^b^ ± 0.71	80.82 ^a^ ± 2.20	65.68 ^b^ ± 1.79
Lipid profile			
T-Ch (mg/dL)	84.83 ^b^ ± 2.12	113.90 ^a^ ± 3.16	90.83 ^b^ ± 1.72
TG (mg/dL)	76.22 ^b^ ± 3.74	130.49 ^a^ ± 2.57	88.50 ^b^ ± 1.84
HDL-Ch (mg/dL)	44.33 ^a^ ± 0.49	26.50 ^b^ ± 0.76	43.00 ^a^ ± 0.52
LDL-Ch (mg/dL)	25.26 ^b^ ± 2.78	61.30 ^a^ ± 3.63	30.13 ^b^ ± 1.81
Oxi-LDL (pg/mL)	27.50 ^c^ ± 0.76	52.50 ^a^ ± 1.12	35.67 ^b^ ± 1.09
Liver and kidney functions			
AST (IU/L)	35.83 ^b^ ± 3.60	62.00 ^a^ ± 3.16	37.33 ^b^ ± 4.68
ALT (IU/L)	5.33 ^b^ ± 0.84	10.00 ^a^ ± 0.89	5.67 ^b^ ± 0.80
Urea (mg/dL)	49.18 ^b^ ± 1.75	58.96 ^a^ ± 1.14	50.72 ^b^ ± 1.59
Creatinine (mg/dL)	0.65 ^b^ ± 0.02	0.98 ^a^ ± 0.08	0.64 ^b^ ± 0.02
Nutritional parameters			
Initial Weight (g)	105.67 ^a^ ± 1.48	105.67 ^a^ ± 1.84	106.17 ^a^ ± 2.21
Final Weight (g)	167.2 ^a^ ± 2.87	145.7 ^b^ ± 1.56	162.2 ^a^ ± 4.20
Body Weight Gain (g)	61.5 ^a^ ± 3.66	40.00 ^b^ ± 2.73	56.00 ^a^ ± 2.78
Spleen %	0. 70 ^b^ ± 0.01	0.98 ^a^ ± 0.03	0.71 ^b^ ± 0.03
Liver %	3.22 ^a^ ± 0.12	3.93 ^a^ ± 0.17	3.33 ^a^ ± 0.13

Values are mean ± SE, *n* = 6. Within the same row, means with different letters (a, b and c) show significant difference between values at a probability level of 0.05. NC: normal control; ARC: arthritic control; APN: avocado peel nutraceutical.

**Table 6:** ∆G Binding affinity (kcal/mol) for each ligand with PTPN22.

Name of the Ligand	Binding Free Energy (kcal/mol)
Ellagic acid	−9.2
β-amyrine	−8.6
Quercetin	−8.4
Naringenin	−8.2
Campesterol	−8.1
Hesperetin	−8
Kaempferol	−8
Stigmasterol	−8
β-Sitosterol	−8
Daidzein	−7.5
Rosmarinic	−7.5
Chlorogenic	−6.7
Ferulic acid	−6.6
Caffeic acid	−6.4
Cinnamic acid	−5.9
Coumaric acid	−5.8
Gallic acid	−5.8
Methyl gallate	−5.8
Vanillin	−5.5
Syringic acid	−5.5
Linoleic acid	−5.2
Oleic acid	−5.1
Pyro-chatechol	−4.8
Linolenic acid	−4.8
Benzaldehyde	−4.7
Ethyl hexanoate	−4.7
Palmitic	−4.5
2-Furfural	−4.4
Decanal	−4.3
Isobutyl propanoate	−4.1
Pentanol	−4.1
Hexanal	−3.6
Ethyl acetate	−3.2
Methyl propanoate	−3.2
Methional	−3.2

**Table 7:** 3D and 2D interactions between each compound with protein tyrosine phosphatase non-receptor type 22.

Ligands	PTPN22	
	2D	3D
Ellagic acid	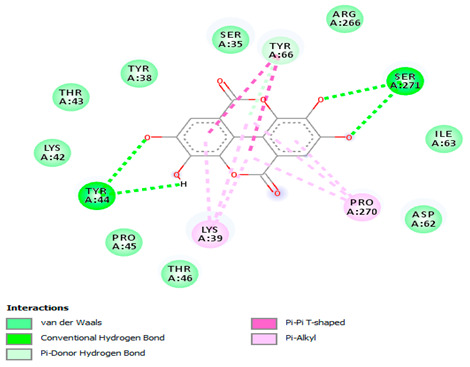	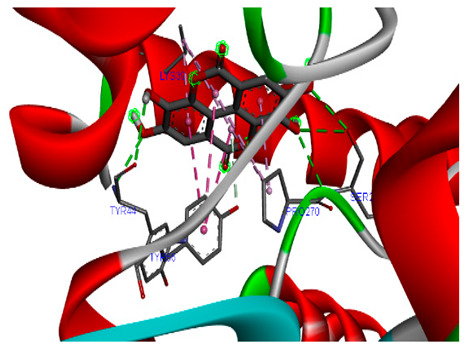
Quercetin	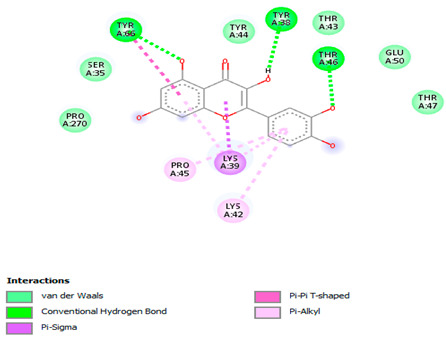	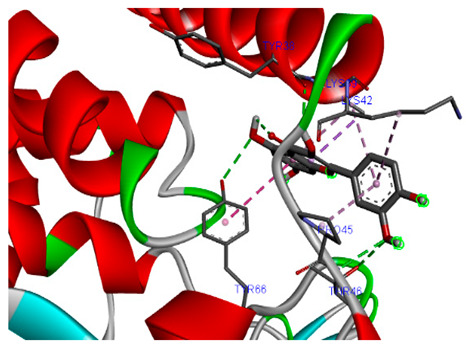
Naringenin	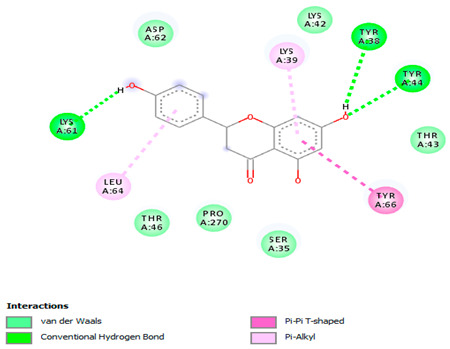	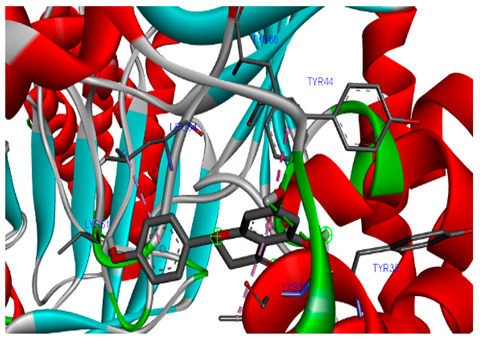
β-amyrine	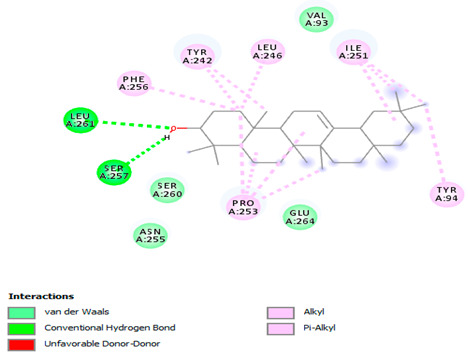	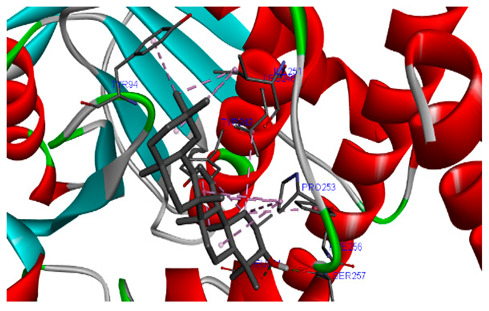
Campesterol	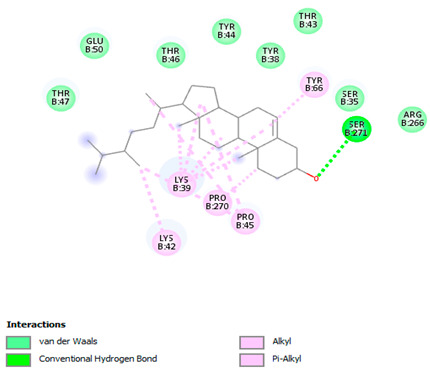	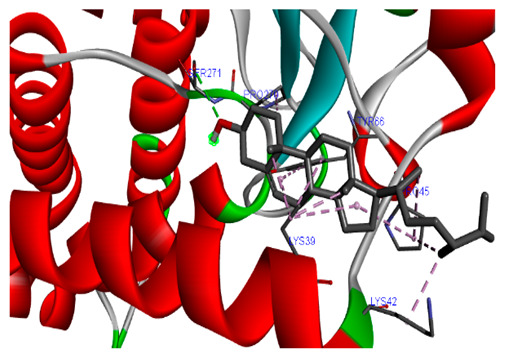
β-Sitosterol	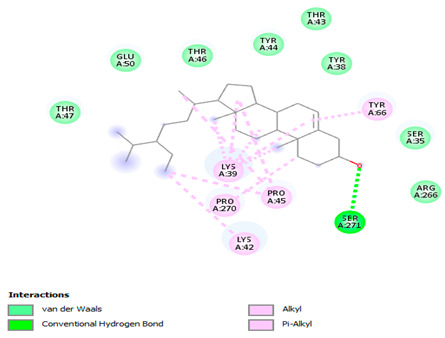	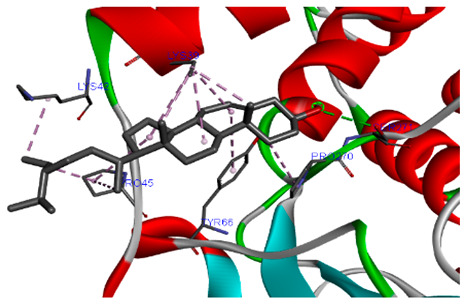
Stigmasterol	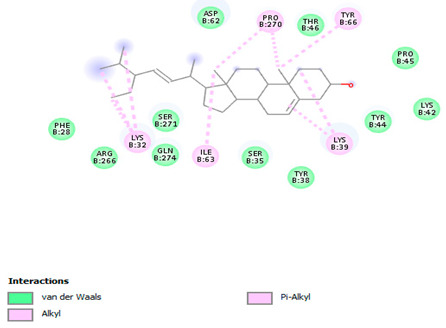	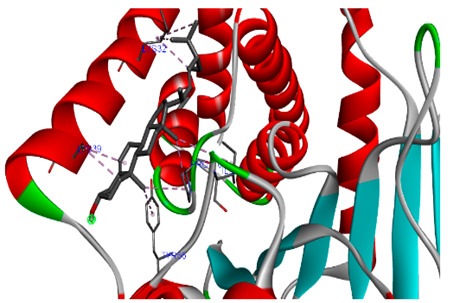

## Data Availability

All data generated or analyzed during this study are included in this published article.

## References

[1] Monteleone G., Moscardelli A., Colella A., Marafini I., Salvatori S. (2023). Immune-mediated inflammatory diseases: Common and different pathogenic and clinical features. Autoimmun. Rev..

[2] Liang Y., Liu M., Cheng Y., Wang X., Wang W. (2023). Prevention and treatment of rheumatoid arthritis through traditional Chinese medicine: Role of the gut microbiota. Front. Immunol..

[3] Brown J.M., Hazen S.L. (2015). The gut microbial endocrine organ: Bacterially derived signals driving cardiometabolic diseases. Annu. Rev. Med..

[4] Yang Y., Hong Q., Zhang X., Liu Z. (2024). Rheumatoid arthritis and the intestinal microbiome: Probiotics as a potential therapy. Front. Immunol..

[5] Attur M., Scher J.U., Abramson S.B., Attur M. (2022). Role of Intestinal Dysbiosis and Nutrition in Rheumatoid Arthritis. Cells.

[6] Sun X., Qian Y., Cheng W., Ye D., Liu B., Zhou D., Wen C., Andreassen O.A., Mao Y. (2024). Characterizing the polygenic overlap and shared loci between rheumatoid arthritis and cardiovascular diseases. BMC Med..

[7] Figus F.A., Piga M., Azzolin I., McConnell R., Iagnocco A. (2021). Rheumatoid arthritis: Extra-articular manifestations and comorbidities. Autoimmun. Rev..

[8] Yang W., Cong Y. (2021). Gut microbiota-derived metabolites in the regulation of host immune responses and immune-related inflammatory diseases. Cell Mol. Immunol..

[9] Lin L., Zhang K., Xiong Q., Zhang J., Cai B., Huang Z., Yang B., Wei B., Chen J., Niu Q. (2023). Gut microbiota in pre-clinical rheumatoid arthritis: From pathogenesis to preventing progression. J. Autoimmun..

[10] Luo Y., Tong Y., Wu L., Niu H., Li Y., Su L.C., Wu Y., Bozec A., Zaiss M.M., Qing P. (2023). Alteration of gut microbiota in individuals at high-Risk for rheumatoid arthritis associated with disturbed metabolome and the initiation of arthritis through the triggering of mucosal immunity imbalance. Arthritis Rheumatol..

[11] Wang Q., Zhang S.-X., Chang M.-J., Qiao J., Wang C.-H., Li X.-F., Yu Q., He P.-F. (2022). Characteristics of the gut microbiome and its relationship with peripheral cd4+ T cell subpopulations and cytokines in rheumatoid arthritis. Front. Microbiol..

[12] Gomez A., Luckey D., Yeoman C.J., Marietta E.V., Berg Miller M.E., Murray J.A., White B.A., Taneja V. (2012). Loss of sex and age driven differences in the gut microbiome characterize arthritis-susceptible 0401 mice but not arthritis-resistant 0402 mice. PLoS ONE..

[13] Scher J.U., Abramson S.B. (2013). Periodontal disease, Porphyromonas gingivalis, and rheumatoid arthritis: What triggers autoimmunity and clinical disease?. Arthritis Res. Ther..

[14] Xu X., Wang M., Wang Z., Chen Q., Chen X., Xu Y., Dai M., Wu B., Li Y. (2022). The bridge of the gut-joint axis: Gut microbial metabolites in rheumatoid arthritis. Front. Immunol..

[15] Yu D., Du J., Pu X., Zheng L., Chen S., Wang N., Li J., Chen S., Pan S., Shen B. (2021). The gut microbiome and metabolites are altered and interrelated in patients with rheumatoid arthritis. Front. Cell. Infect. Microbiol..

[16] He J., Chu Y., Li J., Meng Q., Liu Y., Jin J., Wang Y., Wang J., Huang B., Shi L. (2022). Intestinal butyrate-metabolizing species contribute to autoantibody production and bone erosion in rheumatoid arthritis. Sci. Adv..

[17] Sayago-Ayerdi S., García-Martínez D.L., Ramírez-Castillo A.C., Ramírez-Concepción H.R., Viuda-Martos M. (2021). Tropical Fruits and Their Co-Products as Bioactive Compounds and Their Health Effects: A Review. Foods.

[18] Izu G.O., Mfotie Njoya E., Tabakam G.T., Nambooze J., Otukile K.P., Tsoeu S.E., Fasiku V.O., Adegoke A.M., Erukainure O.L., Mashele S.S. (2024). Unravelling the Influence of Chlorogenic Acid on the Antioxidant Phytochemistry of Avocado (*Persea americana* Mill.) Fruit Peel. Antioxidants.

[19] Ejiofor N.C., Ezeagu I.E., Ayoola M.B., Umera E.A. (2018). Determination of the Chemical Composition of Avocado (*Persea americana*) Seed. Adv. Food Technol. Nutr. Sci. SE.

[20] Martínez-Gutiérrez E. (2023). Study of Influence of Extraction Method on the Recovery Bioactive Compounds from Peel Avocado. Molecules.

[21] Tesfaye T., Ayele M., Gibril M., Ferede E., Limeneh D.Y., Kong F. (2022). Beneficiation of avocado processing industry by-product: A review on future prospect. Curr. Res. Green Sustain. Chem..

[22] Rodríguez-Sánchez D., Silva-Platas C., Rojo R.P., García N., Cisneros-Zevallos L., García-Rivas G., Hernández-Brenes C. (2013). Activity-guided identification of acetogenins as novel lipophilic antioxidants present in avocado pulp (*Persea americana*). J. Chromatogr. B Anal. Technol. Biomed. Life Sci..

[23] Donoso C., Raluca M.A., Chávez-Jinez S., Vera E. (2024). Hass Avocado (*Persea americana* Mill.) Peel Extract Reveals Antimicrobial and Antioxidant Properties against Verticillium theobromae, Colletotrichum musae, and Aspergillus niger Pathogens Affecting Musa acuminata Colla Species, in Ecuador. Microorganisms.

[24] Bhuyan D.J., Alsherbiny M.A., Perera S., Low M., Bas A., Devi O.A., Barooah M.S., Li C.G., Papoutsis K. (2019). The Odyssey of bioactive compounds in avocado (*Persea americana*) and their health benefits. Antioxidant.

[25] Olas B. (2024). The Pulp, Peel, Seed, and Food Products of *Persea americana* as Sources of Bioactive Phytochemicals with Cardioprotective Properties: A Review. Int. J. Mol. Sci..

[26] Rojas-García A., Fuentes E., Cádiz-Gurrea M.L., Rodriguez L., Villegas-Aguilar M.D.C., Palomo I., Arráez-Román D., Segura-Carretero A. (2022). Biological Evaluation of Avocado Residues as a Potential Source of Bioactive Compounds. Antioxidants.

[27] Velioglu Y.S., Mazza G., Gao L., Oomah B.D. (1998). Antioxidant activity and total phenolics in selected fruits, vegetables and grain products. J. Agri Food Chem..

[28] Zhishen J., Mencheng T., Jianming W. (1999). The determination of flavonoid contents in mulberry and their scavenging effect on superoxide radicals. Food Chem..

[29] Mohamed D.A., Mabrok H.B., Ramadan A.A., Elbakry H.F. (2024). Alkaline Diets Potential Role in Prevention of Kidney Stone Formation: In-vivo and Molecular Docking Studies. Food Funct..

[30] Hussein A., Ibrahim G., Kamil M., El-Shamarka M., Mostafa S., Mohamed D.A. (2021). Spirulina-Enriched Pasta as Functional Food Rich in Protein and Antioxidant. Biointerface Res. Appl. Chem..

[31] Adams R. (2007). Identification of Essential Oil Components by Gas Chromatography/Mass Spectrometry.

[32] AOAC (2023). Official Methods of Analysis of the Association of Official Analytical Chemists.

[33] Mohamed D.A., Hamed I.M., Mohammed S.E. (2021). Utilization of grape and apricot fruits by-products as cheap source for biologically active compounds for health promotion. Egypt. J. Chem..

[34] Fatemi S.H., Hammond E.G. (1980). Analysis of Oleate, Linoleate and Linolenate Hydroperoxides in Oxidized Ester Mixtures. Lipids.

[35] Ulbricht T.L.V., Southgate D.A.T. (1991). Coronary Heart Disease: Seven Dietary Factors. Lancet.

[36] Brand-Williams W., Cuvelier M., Berset C. (1995). Use of a free redical method to evaluate antioxidant activity. LWT—Food Sci. Technol..

[37] Re R., Pellegrini N., Proteggente A., Pannala A., Yang M., Rice-Evans C. (1999). Antioxidant activity applying an improved ABTS radical cation decolorization assay. Free Radic. Biol. Med..

[38] Hassan M., Abdel-Moniem S., Mahmoud E.A., Mohamed D.A. (2022). Antioxidant, anti-cancer and anti-arthritic activities of acetogenin-rich extract of avocado pulp. Egypt. J. Chem..

[39] Satoh K. (1978). Serum lipid peroxide in cerebrovascular disorders determined by a new colorimetric method. Clin. Chim. Acta.

[40] Aebi H. (1984). Catalase in vitro. Methods Enzym..

[41] Livak K.J., Schmittgen T.D. (2001). Analysis of relative gene expression data using real-time quantitative PCR and the 2^−ΔΔCt^ method. Methods.

[42] Chun J.M., Kim H.S., Lee A.Y., Kim S.H., Kim H.K. (2016). Anti-inflammatory and anti-osteoarthritis effects of *Saposhnikovia divaricata* ethanol extract: In vitro and in vivo studies. Evid. Based Complement. Altern. Med..

[43] Khan H.A., Abdelhalim M.K., Alhomida A.S., Al Ayed M.S. (2013). Transient increase in IL-1β, IL-6 and TNF-α gene expression in rat liver exposed to gold nanoparticles. Genet. Mol. Res..

[44] Edgar Robert C. (2013). UPARSE: Highly accurate OTU sequences from microbial amplicon reads. Nat. Methods.

[45] Bokulich N.A., Subramanian S., Faith J.J., Gevers D., Gordon J.I., Knight R., Mills D.A., Caporaso J.G. (2013). Quality-filtering vastly improves diversity estimates from Illumina amplicon sequencing. Nat. Methods.

[46] Kebede B., Ting V., Eyres G., Oey I. (2020). Volatile changes during storage of shelf stable Apple juice: Integrating GC-MS fingerprinting and chemometrics. Foods.

[47] Komthong P., Noriyuki I., Mitsuya S. (2007). Effect of ascorbic acid on the odours of cloudy apple juice. Food Chem..

[48] Abdel-Moein N.M., Abdel-Moniem E.A., Mohamed D.A., Hanfy E.A. (2011). Evaluation of the Anti-inflammatory and Anti-arthritic Effects of Some Plants Extracts. Grasas y Aceites.

[49] Al-Okbi S.Y., Mohamed D.A., Kandil E., Abo-Zeid M.A., Mohammed S.E., Ahmed E.K. (2017). Anti-inflammatory activity of two varieties of pumpkin seed oil in an adjuvant arthritis model in rats. Grasas y Aceites.

[50] Mohamed D.A., Hanfy E.A., Fouda K. (2018). Evaluation of Antioxidant, Anti-inflammatory and Anti-arthritic Activities of Yarrow (*Achillea millefolium*). J. Biol. Sci..

[51] Liu N., Fan X., Shao Y., Chen S., Wang T., Yao T., Chen X. (2024). Resveratrol attenuates inflammation and fibrosis in rheumatoid arthritis-associated interstitial lung disease via the AKT/TMEM175 pathway. J. Transl. Med..

[52] Ranjbar M., Shab-Bidar S., Rostamian A., Mohammadi H., Djafarian K. (2024). The effects of intermittent fasting diet on quality of life, clinical symptoms, inflammation, and oxidative stress in overweight and obese postmenopausal women with rheumatoid arthritis: Study protocol of a randomized controlled trial. Trials.

[53] Rutz S., Ouyang W. (2016). Regulation of Interleukin-10 Expression. Adv. Exp. Med. Biol..

[54] Laragione T., Harris C., Azizgolshani N., Beeton C., Bongers G., Gulko P.S. (2023). Magnesium increases numbers of Foxp3+ Treg cells and reduces arthritis severity and joint damage in an IL-10-dependent manner mediated by the intestinal microbiome. EBioMedicine.

[55] Pandolfi F., Franza L., Carusi V., Altamura S., Andriollo G., Nucera E. (2020). Interleukin-6 in Rheumatoid Arthritis. Int. J. Mol. Sci..

[56] Kumar R., Singh S., Saksena A., Pal R., Jaiswal R., Kumar R. (2019). Effect of *Boswellia Serrata* extract on acute inflammatory parameters and tumor necrosis factor-α in complete Freund’s adjuvant-induced animal model of rheumatoid arthritis. Int. J. Appl. Basic Med. Res..

[57] Jang D.I., Lee A.H., Shin H.Y., Song H.R., Park J.H., Kang T.B., Lee S.R., Yang S.H. (2021). The Role of Tumor necrosis factor alpha (TNF-α) in autoimmune disease and current TNF-α inhibitors in therapeutics. Int. J. Mol. Sci..

[58] Al-Roub A., Al Madhoun A., Akhter N., Thomas R., Miranda L., Jacob T., Al-Ozairi E., Al-Mulla F., Sindhu S., Ahmad R. (2021). IL-1β and TNFα Cooperativity in Regulating IL-6 Expression in Adipocytes Depends on CREB Binding and H3K14 Acetylation. Cells.

[59] York A.G., Skadow M.H., Oh J., Qu R., Zhou Q.D., Hsieh W.Y., Mowel W.K., Brewer J.R., Kaffe E., Williams K.J. (2024). IL-10 constrains sphingolipid metabolism to limit inflammation. Nature.

[60] Ouyang W., O’Garra A. (2019). IL-10 Family Cytokines IL-10 and IL-22: From Basic Science to Clinical Translation. Immunity.

[61] Chen Y., Xue R., Jin X., Tan X. (2018). Antiarthritic activity of diallyl disulfide against Freund’s adjuvant-induced arthritic rat model. J. Environ. Pathol. Toxicol. Oncol..

[62] Chen G., Song Y., Ma F., Ma Y. (2020). Anti-arthritic activity of D-carvone against complete Freund’s adjuvant-induced arthritis in rats through modulation of inflammatory cytokines. Korean J. Physiol. Pharmacol..

[63] Sanghavi N., Ingrassia J.P., Korem S., Ash J., Pan S., Wasserman A. (2024). Cardiovascular Manifestations in Rheumatoid Arthritis. Cardiol. Rev..

[64] Yan J., Yang S., Han L., Ba X., Shen P., Lin W., Li T., Zhang R., Huang Y., Huang Y. (2023). Dyslipidemia in rheumatoid arthritis: The possible mechanisms. Front Immunol..

[65] Popescu D., Rezus E., Badescu M.C., Dima N., Seritean Isac P.N., Dragoi I.T., Rezus C. (2023). Cardiovascular Risk Assessment in Rheumatoid Arthritis: Accelerated Atherosclerosis, New Biomarkers, and the Effects of Biological Therapy. Life.

[66] Jomova K., Raptova R., Alomar S.Y., Alwasel S.H., Nepovimova E., Kuca K., Valko M. (2023). Reactive oxygen species, toxicity, oxidative stress, and antioxidants: Chronic diseases and aging. Arch. Toxicol..

[67] Calderón-Oliver M., Escalona-Buendía H.B., Medina-Campos O.N., Pedraza-Chaverri J., Pedroza-Islas R., Ponce-Alquicira E. (2016). Optimization of the antioxidant and antimicrobial response of the combined effect of nisin and avocado byproducts. LWT Food Sci. Technol..

[68] López-Cobo A., Gómez-Caravaca A.M., Pasini F., Caboni M.F., Segura-Carretero A., Fernández-Gutiérrez A. (2016). HPLC-DAD-ESI QTOF-MSandHPLC-FLD-MSasvaluable tools for the determination of phenolic and other polar compounds in the edible part and by-products of avocado. LWT.

[69] Naveed M., Hejazi V., Abbas M., Kamboh A.A., Khan G.J., Shumzaid M., XiaoHui Z. (2018). Chlorogenic acid (CGA): A pharmaco logical review and call for further research. Biomed. Pharmacother..

[70] Tremocoldi M.A., Rosalen P.L., Franchin M., Massarioli A.P., Denny C., Daiuto É.R., Paschoal J.A.R., Melo P.S., Alencar S.M. (2018). Exploration of avocado by-products as natural sources of bioactive compounds. PLoS ONE.

[71] Mohamed D.A., Ismael A.I., Ibrahim A.R. (2005). Studying the anti-inflammatory and biochemical effects of wheat germ oil. Dtsch. Leb. Rundsch..

[72] Jayedi A., Shab-Bidar S. (2020). Fish consumption and the risk of chronic disease: An umbrella review of meta-analyses of prospective cohort studies. Adv. Nutr..

[73] Mohamed D.A., Mohammed S.E., Hamed I.M. (2022). Chia seeds oil enriched with phytosterols and mucilage as a cardioprotective dietary supplement towards inflammation, oxidative stress, and dyslipidemia. J. Herbmed. Pharmacol..

[74] Salazar-López N.J., Domínguez-Avila J.A., Yahia E.M., Belmonte-Herrera B.H., Wall-Medrano A., Montalvo-González E., González-Aguilar G.A. (2020). Avocado fruit and by-products as potential sources of bioactive compounds. Food Res. Int..

[75] Alkhalaf M., Alansari W., Ibrahim E., ELhalwagy M. (2019). Antioxidant, anti-inflammatory and anti-cancer activities of avocado (*Persea americana*) fruit and seed extract. J. King Saud Univ. Sci..

[76] Rosero J.C., Cruz S., Osorio C., Hurtado N. (2019). Analysis of Phenolic Composition of Byproducts (Seeds and Peels) of Avocado (*Persea americana* Mill.) Cultivated in Colombia. Molecules.

[77] Hammad S., Pu S., Jones P.J. (2016). Current Evidence Supporting the Link between Dietary Fatty Acids and Cardiovascular Disease. Lipids.

[78] Cervantes-Paz B., Yahia E.M. (2021). Avocado oil: Production and market demand, bioactive components, implications in health, and tendencies and potential uses. Compr. Rev. Food Sci. Food Saf..

[79] Marović R., Badanjak Sabolović M., Brnčić M., Ninčević Grassino A., Kljak K., Voća S., Karlović S., Rimac Brnčić S. (2024). The Nutritional Potential of Avocado By-Products: A Focus on Fatty Acid Content and Drying Processes. Foods.

[80] Khalili Tilami S., Kouřimská L. (2022). Assessment of the Nutritional Quality of Plant Lipids Using Atherogenicity and Thrombogenicity Indices. Nutrients.

[81] Rabiej-Kozioł D., Momot-Ruppert M., Stawicka B., Szydłowska-Czerniak A. (2023). Health Benefits, Antioxidant Activity, and Sensory Attributes of Selected Cold-Pressed Oils. Molecules.

[82] Kinashi Y., Hase K. (2021). Partners in Leaky Gut Syndrome: Intestinal Dysbiosis and Autoimmunity. Front. Immunol..

[83] Horta-Baas G., Romero-Figueroa M.D.S., Montiel-Jarquín A.J., Pizano-Zárate M.L., García-Mena J., Ramírez-Durán N. (2017). Intestinal Dysbiosis and Rheumatoid Arthritis: A Link between Gut Microbiota and the Pathogenesis of Rheumatoid Arthritis. J. Immunol. Res..

[84] Zhao Y., Cheng M., Zou L., Yin L., Zhong C., Zha Y., Zhu X., Zhang L., Ning K., Han J. (2022). Hidden link in gut–joint axis: Gut microbes promote rheumatoid arthritis at early stage by enhancing ascorbate degradation. Gut.

[85] Zhang Y., Zhen S., Xu H., Sun S., Wang Z., Li M., Zou L., Zhang Y., Zhao Y., Cui Y. (2024). Vitamin C alleviates rheumatoid arthritis by modulating gut microbiota balance. Biol. Sci. Trends.

[86] Chen J., Wright K., Davis J.M., Jeraldo P., Marietta E.V., Murray J., Nelson H., Matteson E.L., Taneja V. (2016). An expansion of rare lineage intestinal microbes characterizes rheumatoid arthritis. Genome Med..

[87] Wang X., Wei G., Ding Y., Gui X., Tong H., Xu X., Zhang S., Sun Z., Ju W., Li Y. (2022). Protein tyrosine phosphatase PTPN22 negatively modulates platelet function and thrombus formation. Blood.

[88] Lee J.Y., Mannaa M., Kim Y., Kim J., Kim G.T., Seo Y.S. (2019). Comparative Analysis of Fecal Microbiota Composition Between Rheumatoid Arthritis and Osteoarthritis Patients. Genes.

[89] Manco M., Putignani L., Bottazzo G.F. (2010). Gut microbiota, lipopolysaccharides, and innate immunity in the pathogenesis of obesity and cardiovascular risk. Endocr. Rev..

[90] Han S.K., Shin Y.J., Lee D.Y., Kim K.M., Yang S.J., Kim D.S., Choi J.W., Lee S., Kim D.H. (2021). *Lactobacillus rhamnosus* HDB1258 modulates gut microbiota-mediated immune response in mice with or without lipopolysaccharide-induced systemic inflammation. BMC Microbiol..

[91] Liu X., Zou Q., Zeng B., Fang Y., Wei H. (2013). Analysis of Fecal *Lactobacillus* Community Structure in Patients with Early Rheumatoid Arthritis. Curr. Microbiol..

[92] Liu Y., Zhang Y., Zhang J., Ren S., Cao Q., Kong H., Xu Q., Liu R. (2024). High-fat diet stimulated butyric acid metabolism dysbiosis, altered microbiota, and aggravated inflammatory response in collagen-induced arthritis rats. Nutr. Metab..

[93] Gámez-Macías P.E., Félix-Soriano E., Samblas M., Sáinz N., Moreno-Aliaga M.J., González-Muniesa P. (2024). Intestinal Permeability, Gut Inflammation, and Gut Immune System Response Are Linked to Aging-Related Changes in Gut Microbiota Composition: A Study in Female Mice. J. Gerontol. A Biol. Sci. Med. Sci..

[94] Hamada K., Isobe J., Hattori K., Hosonuma M., Baba Y., Murayama M., Narikawa Y., Toyoda H., Funayama E., Tajima K. (2023). *Turicibacter* and *Acidaminococcus*predict immune-related adverse events and efficacy of immune checkpoint inhibitor. Front. Immunol..

[95] Lynch J.B., Gonzalez E.L., Choy K., Faull K.F., Jewell T., Arellano A., Liang J., Yu K.B., Paramo J., Hsiao E.Y. (2023). Gut microbiota Turicibacter strains differentially modify bile acids and host lipids. Nat. Commun..

[96] Cao R., Gao T., Yue J., Sun G., Yang X. (2024). Disordered gut microbiome and alterations in metabolic patterns are associated with hypertensive left ventricular hypertrophy. J. Am. Heart Assoc..

[97] Li M., Yang L., Zhao L., Bai F., Liu X. (2022). Comparison of intestinal microbes in noninfectious anterior scleritis patients with and without rheumatoid arthritis. Front. Microbiol..

[98] Yang K., Zhang L., Liao P., Xiao Z., Zhang F., Sindaye D., Xin Z., Tan C., Deng J., Yin Y. (2020). Impact of gallic acid on gut health: Focus on the gut microbiome, immune response, and mechanisms of action. Front. Immunol..

[99] Kasprzak-Drozd K., Oniszczuk T., Stasiak M., Oniszczuk A. (2021). Beneficial effects of phenolic compounds on gut microbiota and metabolic syndrome. Int. J. Mol. Sci..

[100] Matsumura Y., Kitabatake M., Kayano S.I., Ito T. (2023). Dietary phenolic compounds: Their health benefits and association with the gut microbiota. Antioxidants.

[101] Nesci A., Carnuccio C., Ruggieri V., D’Alessandro A., Di Giorgio A., Santoro L., Gasbarrini A., Santoliquido A., Ponziani F.R. (2023). Gut Microbiota and Cardiovascular Disease: Evidence on the Metabolic and Inflammatory Background of a Complex Relationship. Int. J. Mol. Sci..

[102] Taylor K.D., Wood A.C., Rotter J.I., Guo X., Herrington D.M., Johnson W.C., Post W., Tracy R.P., Rich S.S., Malik S. (2024). Metagenomic Study of the MESA: Detection of *Gemella Morbillorum*and association with coronary heart disease. J. Am. Heart Assoc..

[103] Medina G., Vera-Lastra O., Peralta-Amaro A.L., Jiménez-Arellano M.P., Saavedra M.A., Cruz-Domínguez M.P., Jara L.J. (2018). Metabolic syndrome, autoimmunity and rheumatic diseases. Pharmacol. Res..

[104] Bartels L., Pedersen A., Kristensen N., Jepsen P., Vilstrup H., Stengaard-Pedersen K., Dahlerup J. (2019). *Helicobacter pylori* infection is not associated with rheumatoid arthritis. Scand. J. Rheumatol..

[105] Zheng B., Pan F., Shi M., He C., He B., Wang R., Ren G., Yang S., Zhang S. (2024). 2-Monoacylglycerol Mimetic Liposomes to Promote Intestinal Lymphatic Transport for Improving Oral Bioavailability of Dihydroartemisinin. Int. J. Nanomed..

[106] Dimple P., Fatemeh A., Hossein Z. (2010). Intestinal permeability enhancement of levothyroxine sodium by straight chain fatty acids studied in MDCK epithelial cell line. Eur. J. Pharm. Sci..

[107] Gumus C.E., Gharibzahedi M.T. (2021). Yogurts supplemented with lipid emulsions rich in omega-3 fatty acids: New insights into the fortification, microencapsulation, quality properties, and health-promoting effects. Trends Food Sci. Technol..

[108] Machado M., Sousa S.C., Rodríguez-Alcalá L.M., Pintado M., Gomes A.M. (2023). Bigels as Delivery Systems of Bioactive Fatty Acids Present in Functional Edible Oils: Coconut, Avocado, and Pomegranate. Gels.

[109] Deng H., Jiang J., Zhang S., Wu L., Zhang Q., Sun W. (2023). Network pharmacology and experimental validation to identify the potential mechanism of *Hedyotis diffusa* Willd against rheumatoid arthritis. Sci. Rep..

[110] Radu A.-F., Negru P.A., Radu A., Tarce A.G., Bungau S.G., Bogdan M.A., Tit D.M., Uivaraseanu B. (2023). Simulation-Based Research on Phytoconstituents of Embelia ribes Targeting Proteins with Pathophysiological Implications in Rheumatoid Arthritis. Life.

[111] Gong N., Wang L., An L., Xu Y. (2023). Exploring the active ingredients and potential mechanisms of action of sinomenium acutum in the treatment of rheumatoid arthritis based on systems biology and network pharmacology. Front. Mol. Biosci..

[112] Remuzgo-Martínez S., Genre F., Castañeda S., Corrales A., Moreno-Fresneda P., Ubilla B., Mijares V., Portilla V., González-Vela J., Pina T. (2017). Protein tyrosine phosphatase non-receptor 22 and C-Src tyrosine kinase genes are down-regulated in patients with rheumatoid arthritis. Sci. Rep..

[113] Sharp R.C., Beg S.A., Naser S.A. (2018). Polymorphisms in Protein Tyrosine Phosphatase Non-receptor Type 2 and 22 (PTPN2/22) Are Linked to Hyper-Proliferative T-Cells and Susceptibility to Mycobacteria in Rheumatoid Arthritis. Front. Cell. Infect. Microbiol..

[114] Ruiz-Noa Y., Hernández-Bello J., Llamas-Covarrubias M.A., Palafox-Sánchez C.A., Oregon-Romero E., Sánchez-Hernández P.E., Ramírez-Dueñas M.G., Parra-Rojas I., Muñoz-Valle J.F. (2019). PTPN22 1858C>T polymorphism is associated with increased CD154 expression and higher CD4+ T cells percentage in rheumatoid arthritis patients. J. Clin. Lab. Anal..

[115] Spalinger M.R., Lang S., Gottier C., Dai X., Rawlings D.J., Chan A.C., Rogler G., Scharl M. (2017). PTPN22 regulates NLRP3-mediated IL1B secretion in an autophagy-dependent manner. Autophagy.

[116] Alippe Y., Mbalaviele G. (2019). Omnipresence of Inflammasome Activities in Inflammatory Bone Diseases. Semin. Immunopathol..

[117] Pertovaara M., Raitala A., Juonala M., Kähönen M., Lehtimäki T., Viikari J.S., Raitakari O.T., Hurme M. (2007). Autoimmunity and atherosclerosis: Functional polymorphism of PTPN22 is associated with phenotypes related to the risk of atherosclerosis. The Cardiovascular Risk in Young Finns Study. Clin. Exp. Immunol..

[118] Saccucci P., Banci M., Cozzoli E., Neri A., Magrini A., Bottini E., Gloria-Bottini F. (2011). Atherosclerosis and PTPN22: A study in coronary artery disease. Cardiology.

[119] Li S., Luo Z., Su S., Wen L., Xian G., Zhao J., Xu X., Xu D., Zeng Q. (2023). Targeted inhibition of PTPN22 is a novel approach to alleviate osteogenic responses in aortic valve interstitial cells and aortic valve lesions in mice. BMC Med..

